# Role of Toxin ζ and Starvation Responses in the Sensitivity to Antimicrobials

**DOI:** 10.1371/journal.pone.0086615

**Published:** 2014-01-29

**Authors:** Mariangela Tabone, Virginia S. Lioy, Silvia Ayora, Cristina Machón, Juan C. Alonso

**Affiliations:** Departamento de Biotecnología Microbiana, Centro Nacional de Biotecnología, (CNB-CSIC), Madrid, Spain; Centro de Biología Molecular Severo Ochoa (CSIC-UAM), Spain

## Abstract

A fraction of otherwise antimicrobial-sensitive *Bacillus subtilis* cells, called persisters, are phenotypically tolerant of antimicrobial treatment. We report that, independently of *B. subtilis'* growth phase, transient ζ toxin expression induces a dormant state and alters cellular responses so that cells are more sensitive to antimicrobials with different modes of action. This outcome is modulated by fine tuning (p)ppGpp and GTP levels: i) in the presence of low “dysregulated” (p)ppGpp levels (as in *relA* ^−^ cells) hyper-tolerance to both toxin and antimicrobials was observed; ii) physiological or low (p)ppGpp levels (as in the wild-type, *sasA* ^−^, *sasB* ^−^ and *relA* ^−^ *sasA* ^−^ context) show a normal toxin and antimicrobial tolerance; and iii) lower levels (in *relA* ^−^ *sasB* ^−^) or absence of (p)ppGpp (in the *relA* ^−^ *sasA* ^−^ *sasB* ^−^ context), in concert with elevated GTP levels, potentiate the efficacy of both toxin and antimicrobial action, rendering tolerance vulnerable to eradication.

## Introduction

The human and economical costs of antimicrobial “resistance” exceed € 1.5-billon each year [1]. New antimicrobial “resistance” challenges continue to evolve and spread worldwide; very important among them is the trait known as non-inheritable phenotypic tolerance, also called persistence [2]. The molecular bases of antimicrobial persistence are under active investigation in Proteobacteria. The studies of proteobacterial cells with this trait, called persisters, have identified multiple genes involved in this phenomenon, and several of these are components of toxin-antitoxin (TA) modules. It is well documented that expression of proteobacterial type II toxins which halt protein synthesis (e.g., HipA, RelE, MazF) in high-density slow- or non-growing cells shuts down cell proliferation in a large fraction of cells (dormancy) and increase the rate of persisters, resulting in multidrug tolerance (MDT) [3]–[11]. The causes of antimicrobial persistence are poorly understood in bacteria of the Firmicutes phylum. Since Proteobacteria (*e.g.*, *Escherichia coli*) and Firmicutes (*e.g., Bacillus subtilis*) phyla diverged over one billion years ago, it is expected that the analysis of Firmicutes toxins and the comparison with those of Proteobacteria would contribute to our understanding of antimicrobial persistence in a broader range of bacteria.

Toxin ζ and RelE are the most ubiquitous toxins in nature [12]. The target of these two toxins differ: RelE halts protein synthesis by cleaving mRNA in the ribosomal A site, whereas toxin ζ phosphorylates the 3′-OH group of the amino sugar moiety of UDP-N-acetylglucosamine (UNAG), leading to its conversion to unreactive UNAG-3P and thereby affecting cell wall synthesis [13]–[15]. Toxin ζ is widely presented in Firmicutes, therefore the role of this toxin should be analyzed to gain insight into the molecular bases of antimicrobial persistence in bacteria of this phylum. The plasmid- or chromosomal-borne ζ-ε (also termed PezT-A) TA complex consists of two monomeric long-living ζ toxins (half-life ∼80 min) separated by a dimeric short-living ε (ε_2_) antitoxin (half-life ∼16 min) [16], [17]. Conditions that prevent εζ gene expression or promote ε_2_ antitoxin degradation, by a host-encoded protease(s), permit ζ toxin to act freely to block cell proliferation in the large majority of the cells [16]–[20]. At early times of expression, free wt ζ or its short-living variant (ζY83C, half-life ∼28 min) triggers an heterogeneous set of protective responses and alters the expression of ∼2% of total *E. coli* or *B. subtilis* genes rather than showing a bactericidal behavior [17], [20]. Within the first 15 min, ζY83C toxin increases RelA expression, decreases the GTP pool and reduces lipid metabolism and DNA replication without apparent alteration of the *B. subtilis* proteome [17], [20]. Within the 15–60 min interval, ζY83C expression reduces the synthesis of macromolecules, decreases ATP levels, increases the alarmone guanosine (penta)tetraphosphate ([p]ppGpp) pool size, and alters the membrane potential of *B. subtilis* cells [20]. Within the 60–90 min, ζY83C inhibits cell wall biosynthesis [15], [20]. Finally, expression of ζY83C for longer periods (120–240 min) leads to a fraction (20–30%) of the population stained with propidium iodide (suggesting cell death), and a subpopulation of *B. subtilis* cells exhibits non-inheritable toxin tolerance (1–5×10^−5^ survivals) [17], [20]. However, ζY83C toxin expression for longer periods of time (8–16 h) reduces toxin tolerance of *B. subtilis* cells to very low levels (2–6×10^−8^ colony forming units, CFUs) [19].

The interaction of the ε_2_ antitoxin with the ζ toxin inactivate the toxic effect of the latter. The structure of the inactive ζε_2_ζ complex bound to its target, UNAG, is known [13]–[15]. The molecular mechanisms by which ζ toxin induces reversible cessation of *B. subtilis* proliferation (protective dormancy) and by which a minor subpopulation of toxin-sensitive cells becomes tolerant of transient ζ toxin action are poorly understood. Transient ζ toxin expression triggers the synthesis of (p)ppGpp [20], but cells that cannot activate the responses to starvation (e.g., in the null relA [Δ*relA*] context) show an increase in the fraction of cells tolerant of the ζ toxin [20] and of antimicrobials (hyper-tolerance) (this work). Conversely, overexpression of the HipA7 toxin, which also triggers ppGpp synthesis, increases the rate of Ampicillin (Amp) persistence, but the absence of RelA in this bacterium diminishes the high persistent phenotype in *hipA*7 mutant *E. coli* cells [21], [22]. It is worth mentioning that the global transcriptional response to starvation and the physiological role of (p)ppGpp in *E. coli* cells do not explain the mechanism of action of (p)ppGpp and/or GTP (GDP) in *B. subtilis* ([SI] Annex S1 in File S1) [23], [24]. Therefore ζ toxin might be a good candidate to address the mechanism(s) of antimicrobial tolerance and their potential link with the variations in the (p)ppGpp and/or GTP pool in Firmicutes.

In *B. subtilis*, active starvation responses, as the stringent response induced by amino acid starvation, switch the cell metabolism from the growth mode to the survival mode, resulting in slow growth, high (p)ppGpp and low GTP (or GDP) levels (see SI Annex S1 in file S1 and Figure S1 in file S2). *B. subtilis*, as with many other species of the Firmicutes Phylum, possesses three enzymes that control the cellular pool of (p)ppGpp. The bifunctional synthase-hydrolase RelA is the major modulator of the intracellular levels of (p)ppGpp [25]–[27]. In addition, two secondary monofunctional small alarmone synthases (termed SasA, RelP, Sas1 or YwaC and SasB, RelQ, Sas2 or YjbM) contribute to accumulation of unknown (p)ppGpp levels, and fine-tune the (p)ppGpp levels during homeostatic growth in the wt context (Figure S1 in file S1) [25], [27]–[29]. The rapid accumulation of (p)ppGpp during stress conditions is mainly dependent on the RelA enzyme [27], [28]. In the absence of RelA, there is also a poor growth phenotype and this could be due to “dysregulated” undetectable (p)ppGpp levels, by the contribution of the monofunctional SasA and/or SasB synthases (SI Annex S1 in file S1).

In this study, we report that ζ-mediated inhibition of cell proliferation alters the cell physiology, leading to increased multidrug sensitivity (MDS) rather than MDT. Subsequent expression of the ε_2_ antitoxin specifically reverses ζ-induced inhibition of cell proliferation (dormancy), but not the sensitivity to different antimicrobials. Our results suggest that ζ-induced dormancy *per se* is not sufficient to trigger MDT at least under the experimental conditions used. Fluctuations in (p)ppGpp and/or GTP levels, however, lead to a different response to ζ toxin and antimicrobials. Hyper-tolerance of toxin and antimicrobial action was observed in the *relA^−^* context (“dysregulated” undetectable (p)ppGpp levels). An artificial decrease of (p)ppGpp levels, by transient exposure to limiting relacin concentrations, reduced hyper-tolerance of *relA^−^* cells to levels similar to *relA* ^+^ or *sasA* ^−^ *sasB* ^−^ cells. Lower or absent [p]ppGpp and elevated GTP levels (as occur in the *relA^−^ sasB^−^* or *relA^−^ sasA^−^ sasB^−^* context) sensitize bacterial cells to antimicrobial and toxin action. An artificial decrease of GTP levels, by transient exposure to decoyinine, reduces cells killing in these backgrounds.

## Materials and Methods

### Bacterial strains and media

The bacterial strains used in this study are described in Table S1 in file S2. To express ζ toxin, two inducible systems, integrated as a unique copy in the chromosomal *amy* locus, were used to mimic the native levels of the toxin [17], [20]. Strains containing the ζY83C toxin variant cassette consist of the ζY83C gene transcribed from a xylose (Xyl)-dependent promoter (*P_xyl_*) under the control of XylR repressor and the *cat* gene conferring resistance to chloramphenicol (Cam^R^) (Table S1 in file S2) [17], [20]. In the absence of Xyl, ζY83C toxin expression did not affect cell viability [20].

Strains containing the wt ζ toxin cassette consist of the wt ζ gene transcribed from the hyper-spank promoter (*P_hsp_*) under the control of the LacI repressor, and the *spc* gene conferring resistance to spectinomycin (*lacI-P_hsp_*ζ *spc*) (Table S1 in file S2) [20]. The strains containing the *lacI-P_hsp_*ζ *spc* cassette also carry in addition the pCB799-borne ε gene (transcribed from *P_xyl_* under the control of XylR repressor) [20]. Low Xyl concentrations were needed to construct the strains containing the wt ζ gene under *P_hsp_* transcriptional control [20]. Addition of low Xyl concentration (0.005%) allowed low levels of the ε_2_ antitoxin expressed from pCB799 plasmid, which titrated out basal levels of ζ toxin and avoided genetic rearrangements [20].

*B. subtilis* Δ*mazF* chromosomal DNA obtained from C. Condon (IBPC, France) was used to transform BG687 (control strain, which lacks the ζY83C toxin cassette) and BG689 (contains the ζY83C toxin cassette) competent cells with selection for erythromycin (Ery) resistance, to produce strains BG1241 and BG1243, respectively (Table S1 in file S2). SPP1 stock phages amplified in BG687 or BG689 cells were used to transduce the control or the ζY83C toxin cassette with selection for Cam^R^ into wt, single (Δ*relA*, Δ*sasA*, Δ*sasB*), double (Δ*sasA* Δ*sasB*, Δ*relA* Δ*sasB*, Δ*relA* Δ*sasA*) and triple mutants (Δ*sasA* Δ*sasB* Δ*relA*) obtained from F. Kawamura (RU, Japan) to produce strains BG1202, BG1203, BG1205, BG1207, BG1211, BG1209, BG1301 and BG1213, respectively (Table S1 in file S2).

Except for Δ*relA* derived mutants, the BG214 isogenic strains were grown in S7 minimal medium (MMS7) supplemented with the required amino acid (methionine and tryptophan) at 50 µg/ml [17]. The isogenic Δ*relA* strains show a “phenotypic auxotrophy” for valine, leucine, isoleucine and threonine, and were also supplemented with these amino acids (at 25 µg/ml) [17], [20]. The S7 medium supplemented with the required amino acids was also termed MMS7.

### Expression levels of the ζY83C or ζ toxin

Depending on the expression system, to moderate-density cells (∼5×10^7^ cells/ml) growing at 37°C in MMS7, Xyl (0.5%) or IPTG (1 mM) was added. Cells were taken at different times after Xyl or IPTG, centrifuged, resuspended in buffer A (50 mM Tris HCl [pH 7.5], 150 mM NaCl, 5% glycerol) and lysed by sonication as described [20]. For Western blotting, extracts containing equal concentrations of protein were separated on 10% SDS-PAGE. Blots were probed with rabbit polyclonal antibodies raised against ζ, which were obtained using standard techniques [16]. Previously it was reported that the level of wt ζ toxin in moderate- and high-density cells expressed from its native context, where the ε_2_ antitoxin was also present, was ∼1,400 ζ monomers/cell [16]. Induction of the *xylR*-*P*_XylA_ζY83C cassette, by addition of 0.5% Xyl to exponentially growing BG689 cells rendered low levels of induction. Expression increased to a plateau with a ζY83C toxin concentration of 300±24 toxin monomers/cell at ∼3 to 5 min, and the steady-state level of the toxin remained for at least 240 min as previously described (this work) [20]. Expression of the *xylR*-*P*_XylA_ζY83C cassette, by addition of 0.5% Xyl in the BG1203 background, increased ζY83C to a plateau of 340±40 toxin monomers/cell, and the steady-state level of the toxin remained for at least 240 min in these strains.

Induction in the *lacI-P_hsp_*ζ *spc* cassette, by addition of 1 mM IPTG to exponentially growing BG1125 cells [20], increased ζ toxin to a plateau concentration of 1,700±125 wt ζ monomers/cell and the steady-state level of ζ remained for at least 240 min. In these strains, the presence of 0.005% Xyl, necessary to avoid genomic rearrangements, accounted for low levels of ε_2_ antitoxin expression [∼150 wt ε_2_ antitoxin/cell]. These toxin concentrations are comparable to the level of wt ζ toxin in its native context (∼1,400 ζ monomers/cell) (this work) [16]. Similar results were observed when the levels of fluorescence of wt ζ fused to GFP (ζ-GFP) were measured *in bulk*, in the presence of ε_2_ antitoxin [19]. The level of ζ-GFP fluorescence was relatively homogeneous, however, they vary significantly in a small cell fraction among the individual cells of an otherwise identical population (data not shown). In <1% of total cells the ζ-GFP levels are significantly higher (2- to 3-fold) than average [18], [19].

### Assay for studies on the effect of ζ expression on the viability of antimicrobial treated cells

The minimal inhibitory concentration (MIC) of the different antimicrobials tested was estimated by exposing 1–3×10^6^ cells/ml for 16 h at 37°C in MMS7 with shaking (240 rpm) to different concentrations of the antimicrobials employed. Except for Ery, which was used at 4 times the MIC (20 µg/ml), the remaining antimicrobials were used at twice the MIC (ampicillin [Amp], 3 µg/ml; ciprofloxacin [Cip], 0.4 µg/ml; and triclosan [Tri], 3 µg/ml). Under conditions of no toxin expression (*i.e*, in the absence of inducer), the presence (BG689, BG1125, BG1145, BG1202) of the ζY83C or ζ gene does not affect the MIC (data not shown).

Cells were grown in MMS7 to mid-exponential phase (∼5×10^7^ cells/ml) or to early stationary phase in MMS7 and then diluted into pre-warmed fresh MMS7 to ∼1×10^9^ or ∼1×10^6^ cells/ml. Expression of the toxin was induced either with 1 mM IPTG (wt ζ toxin cassette) or with 0.5% Xyl (ζY83C toxin cassette) and the antimicrobial was added. After addition of Xyl/IPTG and/or antimicrobial, at 120 or 240 min, the cells were centrifuged and re-suspended with fresh LB medium to remove the inductor (Xyl/IPTG) or the antimicrobial, and the corresponding dilutions were plated on LB agar plates containing glucose (which switches off ζY83C expression) or on LB agar plates containing Xyl to express the ε_2_ antitoxin (ζ toxin cassette), unless otherwise indicated. The survival rate, derived from the number of CFUs obtained in a given condition relative to the CFUs of the non-induced/non-antimicrobial treated control is documented. Except Δ*relA* and Δ*sasA* Δ*sasB* Δ*relA* strains, cells grew in MMS7 with a doubling time of 50–60 min. The doubling time of Δ*relA* and Δ*sasA* Δ*sasB* Δ*relA* increased 1.4- to 1.8-fold when compared to the wt strain. All plates were incubated for 20 h at 37°C, except plates were synthase mutant strains were spread, which were incubated for 40 h at 37°C.

Limiting relacin (1 mM) or decoyinine (100 µg/ml) concentrations were added to early-exponential phase (OD_560_ = 0.1) cells, and cells were allowed to grow to mid-exponential phase (OD_560_ = 0.4, ∼5×10^7^ cells/ml) in MMS7. Expression of the toxin was induced with 0.5% Xyl and Amp was added. At 120 min, the cells were centrifuged and re-suspended in fresh LB to remove the antimicrobial and the inducer and the corresponding dilutions were plated on LB agar plates.

## Results and Discussion

### Experimental design

**Figure 1:**
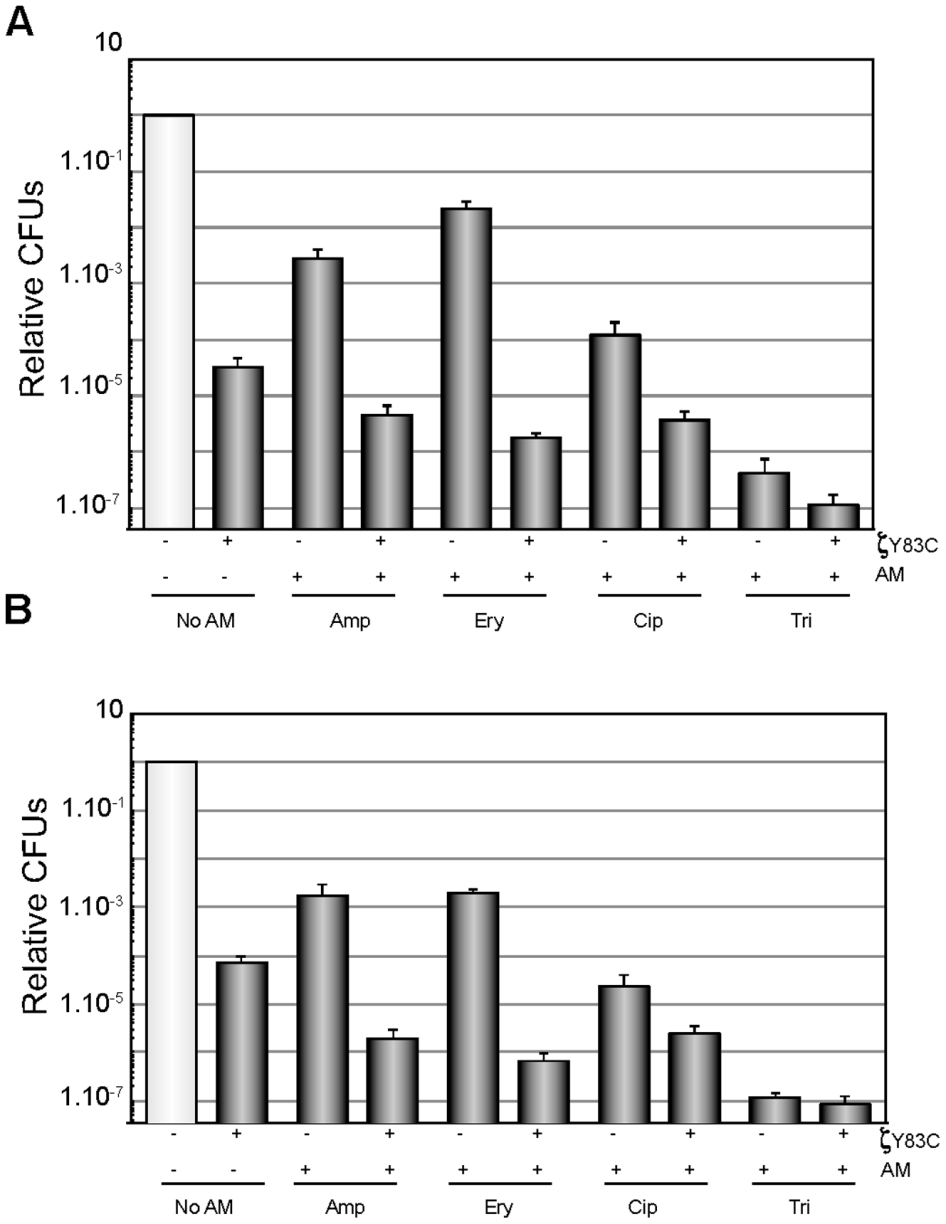
Expression of ζY83C toxin enhances the efficacy of different antimicrobials during exponential growth. BG689 cells were grown in MMS7 at 37°C up to ∼5×10^7^ cells/ml, then 0.5% Xyl and/or an antimicrobial (AM) were added and the cultures were incubated for 120 min A) or 240 min B) and then plated onto LB agar plates. The antimicrobials used were Amp, 3 µg/ml; Ery, 20 µg/ml; Cip, 4 µg/ml or Tri, 3 µg/ml. The number of CFUs relative to the non-induced/non-AM treated control is shown. + and − denote the presence or the absence of the indicated compound. Error bars show 95% confidence intervals of more than three independent experiments.

**Figure 2:**
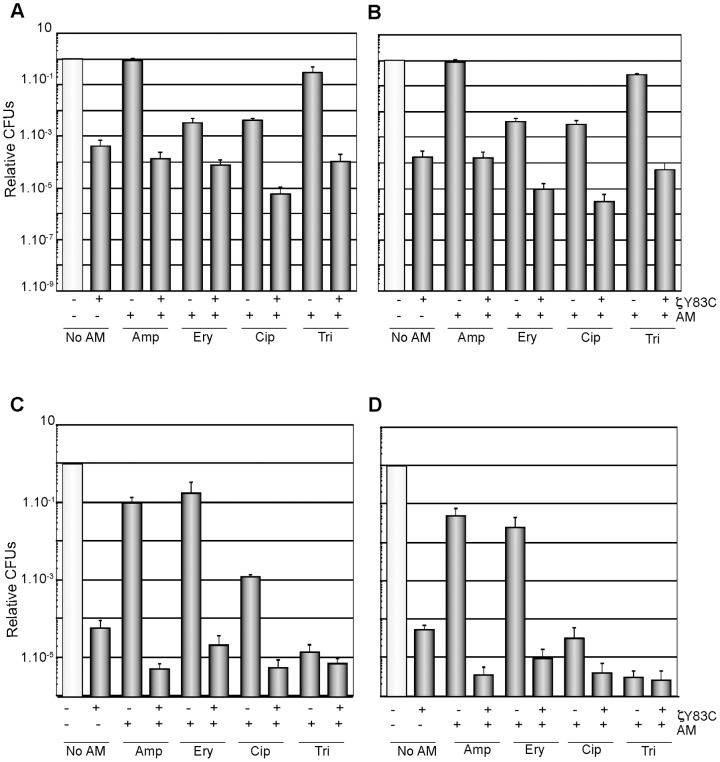
Expression of ζY83C toxin enhances the efficacy of different antimicrobials in high- and low-density non-growing cells. BG689 cells were grown in MMS7 at 37°C up to early stationary phase and diluted into fresh pre-warmed MMS7 to ∼1×10^9^ cells/ml (A and B) or to ∼1×10^6^ cells/ml (C and D). Then 0.5% Xyl and/or an AM were added and the cultures were incubated for 120 min (A and C) or 240 min (B and D). The symbols, the plating conditions, and the antimicrobial concentrations are as described in Figure 1. Error bars show 95% confidence intervals of more than three independent experiments.

**Figure 3:**
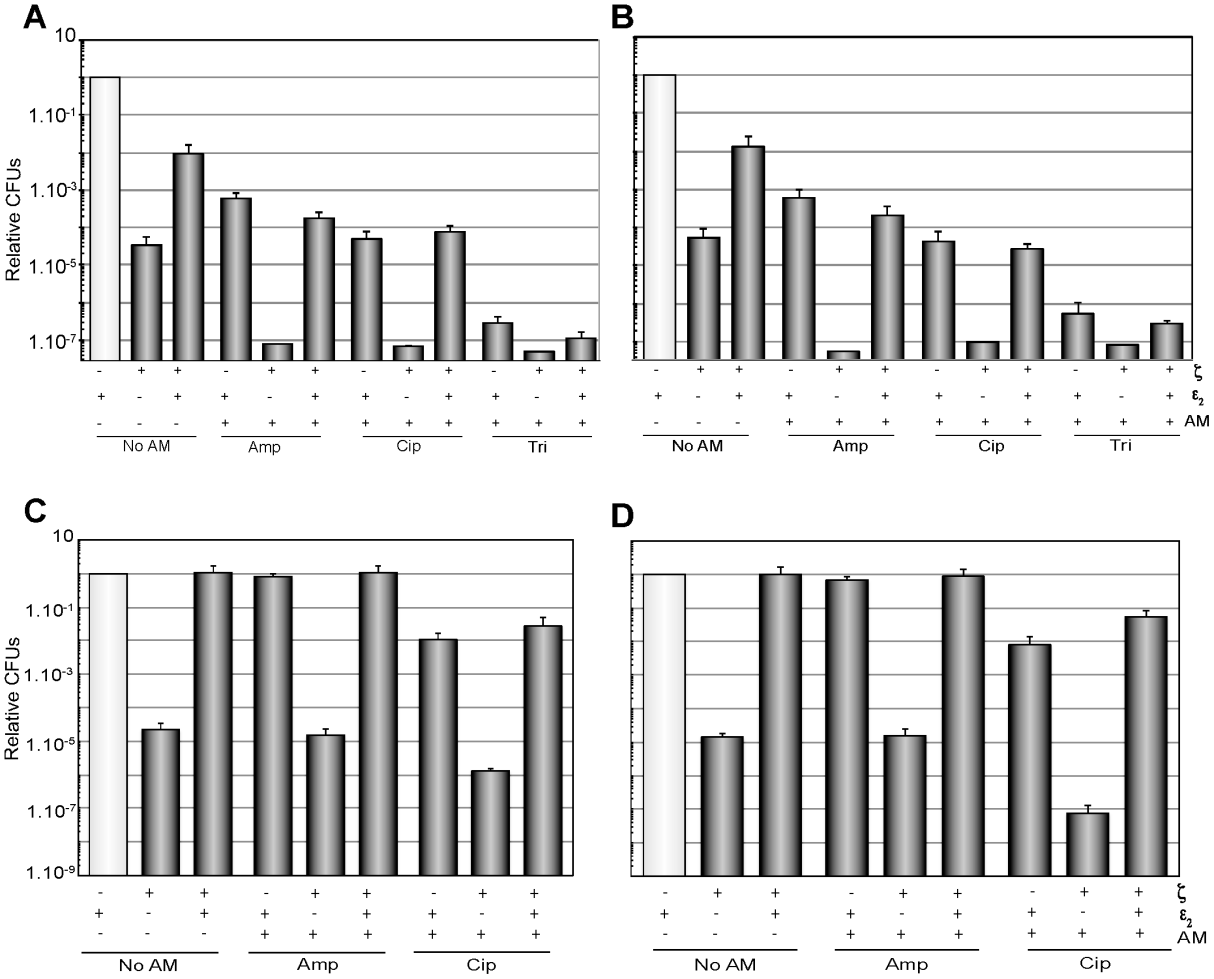
Expression of the ε_2_ antitoxin reverses the effect of ζ toxin, but does not increase MDT. (A and B) BG1125-borne ζ gene was induced by the addition of 1 mM IPTG, and pCB799-borne ε gene was induced by addition of Xyl. BG1125 cells were grown to ∼5×10^7^ cells/ml in MMS7 containing traces of Xyl (0.005%). Then, expression of ζ was induced and/or cells treated with an antimicrobial, and the cultures incubated for 120 min (A) or 240 min (B). At those times, samples were taken and plated on LB agar or on LB-0.5% Xyl plates to induce the expression of the ε_2_ antitoxin. Expression of the ε_2_ antitoxin reverses the effect of ζ toxin in non-growing cells. (C and D) BG1125 cells were grown in MMS7, containing traces of Xyl (0.005%), up to early stationary phase, and diluted to ∼1×10^9^ cells/ml. Then, expression of **ζ** was induced and/or cells treated with an antimicrobial, and the cultures incubated for 120 min (C) or 240 min (D) with agitation at 37°C. At these times samples were taken and plated on LB agar or LB-0.5% Xyl to induce the expression of the ε_2_ antitoxin. The symbols, the plating conditions, and the antimicrobial concentrations were those indicated in Figure 1. Error bars show 95% confidence intervals of more than three independent experiments.

**Figure 4:**
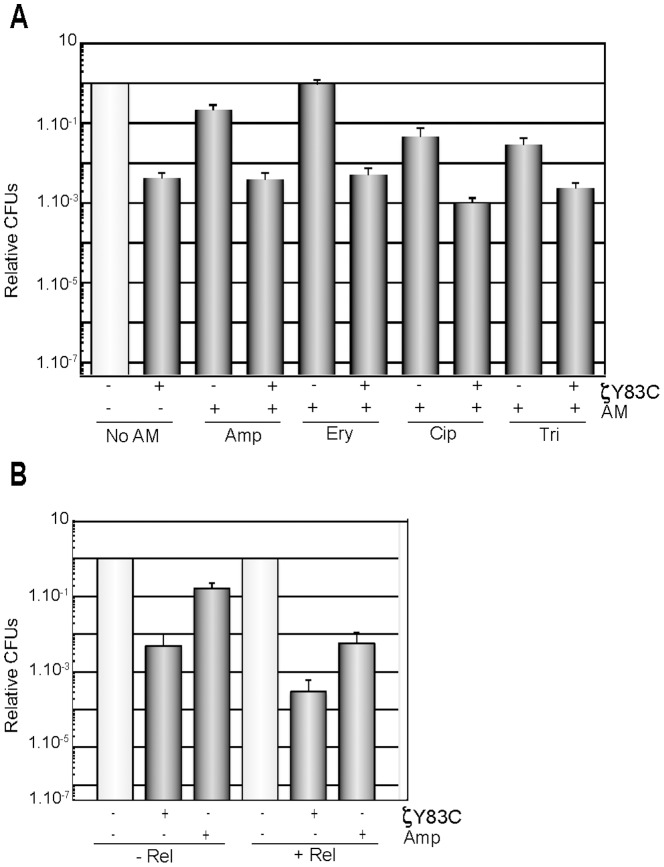
RelA is required for ζY83C toxin enhanced efficacy to different antimicrobials. A) BG1145 cells (Δ*relA*) were grown to ∼5×10^7^ cells/ml. Then 0.5% Xyl and/or an AM were added and the cultures were incubated for 120 min. B) BG1145 cells were pre-treated with limiting relacin (1 mM) concentrations (+ Rel) or not (− Rel), and then 0.5% Xyl, Amp or both were added and the cultures were incubated for 120 min. The symbols, the plating conditions, and the antimicrobial concentrations were those indicated in Figure 1. Error bars show 95% confidence intervals of more than three independent experiments.

**Figure 5:**
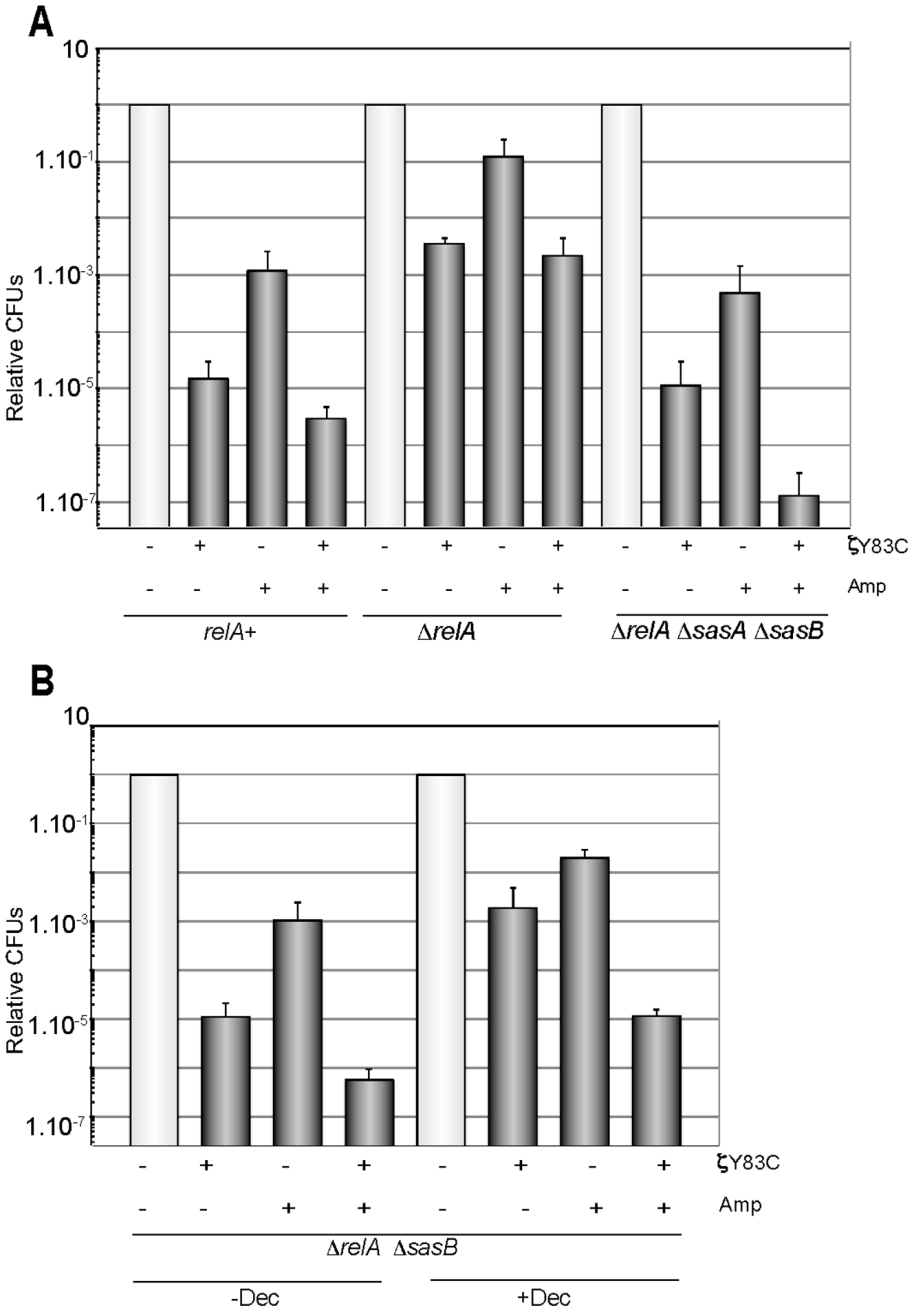
Optimal levels of (p)ppGpp and GTP (GDP) are required for ζY83C and antimicrobial tolerance. A) BG1202 (*relA* ^+^), BG1203 (Δ*relA*) or BG1213 (Δ*relA* Δ*sasA* Δ*sasB*) cells were grown in MMS7 to ∼5×10^7^ cells/ml, then 0.5% Xyl and/or Amp were added and the cultures incubated for 120 min. Appropriate dilutions were then platted on LB agar. B), Δ*relA* Δ*sasB* cells (BG 1209) were pre-treated with decoyinine (100 µg/ml, + Dec) or not (− Dec) for 30 min, and then 0.5% Xyl, Amp or both were added and the cultures were further incubated for 120 min. The symbols, plating conditions, and antimicrobial concentrations were as described in Figure 1. Error bars show 95% confidence intervals of more than three independent experiments.

Antimicrobial persisters (also called tolerants), which might arise through a mechanism analogous to that of toxin tolerance, is a natural multimodal system where a subpopulation of bacterial cells enters a transient-non-growth state that confers tolerance to antimicrobials [7], [11]. The exit from persistence has been linked to the switch between alternative physiological states with cells forming colonies upon plating in a rich medium in the absence of the antimicrobial [11]. At least four types of mechanisms for antimicrobial persistence have been proposed. First, persister bacteria are those that at the time of exposure to the antimicrobials have their targets inactive, as in growth-arrested cells or in a “protected” stage of the cell cycle [11]. Second, toxin action leads to a high frequency of phenotypic persistence to different antimicrobials; leading to MDT [30]. Third, persisters are those able to adapt rapidly to antimicrobial treatment by switching on/off genes linked to general stress responses [31]. Fourth, persistence is a programmed, epigenetic phenomenon with a genetic basis that has evolved to allow prokaryotic organisms to survive changing environments [30], [32]. To test the first two hypotheses we compared the effects of different antimicrobials and controlled ζ toxin expression in exponentially growing wt *B. subtilis* cells, versus stationary phase cells (Figure 1, Figure 2, Figure 3). To test the last two hypotheses, the starvation control mechanisms were disrupted (Figure 4, Figure 5).

### Expression of ζY83C toxin sensitizes cells to antimicrobials during exponential growth

Previously it was shown that low levels of expression of a short-living variant of the ζ toxin (ζY83C), 300 monomers/cell (see Materials and methods), induced dormancy and a condition similar to the effects of amino acid starvation (increasing synthesis of (p)ppGpp, and decreased GTP pool) in the majority of the cells [20]. To test whether toxin-induced dormancy make cells transiently refractory to antimicrobials with different modes of action, moderate-density (∼5×10^7^ cells/ml) exponentially growing cells in MMS7 was used. As already shown, transient expression of the ζY83C toxin under this condition, by addition of 0.5% Xyl (see Materials and Methods), produced typical biphasic survival kinetics with an initial rapid decrease in CFUs in the first 60 min and a tolerant subpopulation with stable CFUs between 60 and 300 min [20], so that for simplicity the data at 120 and 240 min are presented (see Figure 1, Figure 2, Figure 3). A subpopulation of cells were tolerant of ζY83C toxin action, and they were quantified upon serial dilutions and plating in LB agar (2–5×10^−5^ survivals, Figure 1). Alternatively, in this small subpopulation of tolerant cells the ζY83C toxin might not be expressed. To test this hypothesis ζY83C toxin expression was induced for longer periods, and the ratio of tolerant cells was measured. The expression of the ζY83C toxin for 480 or 960 min significantly reduced the tolerant fraction (4–6×10^−8^ survivals, data not shown). Similar results were obtained upon transient expression of the wt ζ toxin for 480 min or longer times, and then inducing the expression of the ε_2_ antitoxin [17]. For this reason we considered unlike that the small subpopulation of ζY83C tolerant cells are simply due to no expression of the toxin. Furthermore, a fused ζ-GFP variant revealed that the level of toxin expression was significantly homogeneous in the majority of the cells [19]. The large majority of these tolerant cells did not genetically acquire resistance to toxin action see [17].

Antimicrobials with different modes of action (bactericidal or bacteriostatic) were selected: Amp inhibits cell wall biosynthesis, Ery inhibits protein translation, Cip inhibits the ligase step of type II topoisomerases, and Tri inhibits fatty acid biosynthesis and triggers (p)ppGpp synthesis, among other targets [33]–[36]. A highly significant variation was observed in persister fractions following exposure to the different antimicrobials, implying that tolerance to an antimicrobial depends on the precise manner in which the antimicrobial acts, and perhaps on the specific mechanism by which the persister phenotype is generated. As observed in Fig. 1A, transient exposure to Amp, Ery or Cip decreased colony formation rendering 2×10^−2^ to 1×10^−4^ survivals upon exposure to the antimicrobial for 120 min. A similar ratio of persistence was observed when the cells were exposed for 60 or 90 min (data not shown). These cells exhibited phenotypic tolerance, because when re-grown, they remained sensitive to the antimicrobial (data not shown). The ratio of persister cells was strongly reduced, to 4–8×10^−7^ survivals, upon transient exposure to Tri for 120 min, and was at the detection limit (∼1×10^−7^) upon exposure to this antimicrobial for 240 min (Figure 1A and 1B). It is likely that bacteria evolved different ways of persisting in response to different antimicrobials, and that a lipid-targeting antimicrobial, such as Tri, might reduce the acquisition of stochastic antimicrobial tolerance in moderate-density exponentially growing cells.

To learn if ζY83C toxin induction may favor the appearance of tolerants, ζY83C toxin was induced at the same time as cells were treated with antimicrobials as described in Materials and Methods. The abundance of ζY83C (∼300 monomers/cell), after Xyl induction, was not significantly affected by the addition of an antimicrobial (e.g., Amp) at least during the first 150 min when compared with absence of the antimicrobial (see Materials and methods). We observed that expression of ζY83C toxin in the presence of an antimicrobial for 120 or 240 min significantly decreased colony formation compared to antimicrobial treatment alone (Figure 1). A similar outcome was observed when dormancy was first induced by addition of Xyl or the antimicrobial 30 min prior challenging by antimicrobial addition or ζY83C toxin expression, respectively (data not shown).

It is likely that: i) ζY83C toxin arrest of cell growth *per se* does not facilitate antimicrobial tolerance, at least with the antimicrobials tested; ii) ζ-induced and stochastic antimicrobial tolerance may be a result of multiple distinct cellular physiologies within a population; iii) the reduction of CFUs upon ζY83C toxin expression and antimicrobial addition may be due to the additive effects of two independent killing mechanisms or ζY83C toxin expression may facilitate the exit of the persister state of different antimicrobials; iv) the sensitization of cells to antimicrobial addition and expression of the ζY83C toxin supports the notion that toxin tolerance cannot be attributed to low or lack of ζY83C expression.

### Toxin ζY83C and Ampicillin trigger different bacterial responses

In the previous section we have shown that: i) expression of the ζY83C toxin, which at latter times inhibits cell wall biosynthesis [15], [20], reduced CFUs of exponentially growing cells ∼50,000-fold, and addition of Cip reduced CFUs ∼10,000-fold; and ii) expression of ζY83C and addition of Cip showed an additive effect, with ∼7×10^−6^ tolerant/ml (Figure 1). To test whether this additive effect was due to the coordinated action of two cell proliferation inhibitors, ζY83C toxin was replaced by another cell wall inhibition (e.g., Amp) and cells were treated with both Amp and Cip simultaneously. Moderate-density (∼5×10^7^ cells/ml) growing cells were transiently exposed to Amp and Cip or both for 120 min. As already observed, the addition of Cip decreased CFUs ∼10,000-fold, and the addition of Amp decreased CFU ∼250-fold (Figure S2 in file S2). However, no additive effect was observed, and transient treatment with both Cip and Amp for 120 min decreased CFUs >2,000-fold, which is lower reduction than when cells were treated only with Cip (Figure S2 in file S2). The data presented in Figure 1 and Figure S2 suggest that expression of ζ toxin triggers specific bacterial responses that might not parallel Amp or Cip responses.

### A host-encoded toxin does not contribute to toxin-mediated antimicrobial sensitivity

Previously it was shown that: i) in exponentially growing *E. coli* cells successive deletion of the ten mRNase-encoding TA loci progressively reduced the level of persisters, and deletion of the ten toxins resulted in up to 200-fold reduction of Amp persisters; and ii) ppGpp, which indirectly activates antitoxin degradation, activates expression of host-encoded toxins that in turn induce the persister-like phenotype [37]. The current knowledge suggests that exponentially growing *B. subtilis* cells express only one mRNase toxin, MazF (also called NdoA) [38], [39]. To test whether the increase in (p)ppGpp levels due to ζY83C toxin expression, or simply if the ζY83C toxin activates expression of the host-encoded MazF toxin, and this putative enhancement of *mazF* expression contributes to antimicrobial persistence, strains bearing a null *mazF* mutation (Δ*mazF*) and the ζY83C toxin expression cassette were constructed (Table S1 in file S2), and analyzed upon addition of Xyl, Amp or both (Table 1). No increase in ζY83C toxin tolerance was observed in *mazF* ^+^ exponentially growing cells when compared with Δ*mazF* (1–2×10^−5^ survivals) (Table 1).

**Table 1:** Effect of a chromosomal-encoded TA locus on the formation of antimicrobial persisters.

strain	Conditions	Toxin^c^	CFUs/ml^d^
*mazF* ^+^	− Xyl	ζY83C^−^	2.2×10^8^
*mazF* ^+^	+ Xyl^a^	ζY83C^+^	2.3×10^3^
*mazF* ^+^	+ Amp^b^	ζY83C^−^	4.5×10^5^
*mazF* ^+^	+ Xyl^a^+Amp^b^	ζY83C^+^	4.4×10^2^
Δ*mazF*	− Xyl	ζY83C^−^	2.4×10^8^
Δ*mazF*	+ Xyl^a^	ζY83C^+^	2.8×10^3^
Δ*mazF*	+ Amp^b^	ζY83C^−^	9.0×10^5^
Δ*mazF*	+ Xyl^a^+Amp^b^	ζY83C^+^	4.6×10^2^

BG689 (ζY83C *mazF* ^+^) and BG1243 (ζY83C Δ*mazF*) cells were grown in MMS7 to ∼5×10^7^ cell ml^−1^. Then 0.5% Xyl^a^ or 2x-MIC Amp^b^ or both was added and the culture was incubated for 120 min.

c Induction or not of the ζY83C toxin is indicated by + and − superscript symbols, respectively.

d The CFUs were measured after 120 min of toxin induction and/or antimicrobial addition by plating appropriate dilutions on LB plates. The results are the average of at least three independent experiments and are within a 10% standard error.

The absence of MazF decreased Amp (2× MIC) lethality by ∼2-fold. Similarly, in a previous report it was shown that in *B. subtilis* the absence of MazF decreased kanamycin (10× MIC) and moxifloxacin (16× MIC) lethality by 10- to 15-fold [39]. When moderate-density (∼5×10^7^ cells/ml) exponentially growing cells were transiently exposed to both Amp and ζY83C toxin action for 120 min the survival rates of *mazF* ^+^ and Δ*mazF* cells were similar (Table 1). Together these data suggested that under the conditions used, the potential activation of MazF (by ζY83C induction of [p]ppGpp synthesis) did not contribute to the increased sensitization to Amp in the presence of ζY83C toxin (Table 1). It is likely that the second hypothesis (see above) might not apply upon ζY83C toxin expression, at least with the antimicrobials used, because the presence or absence the host-encoded *mazF* and ζY83C toxin marginally contribute to persistence.

### Expression of ζY83C toxin sensitizes non-growing cells to antimicrobials

To learn whether nutrient limitation prior to ζY83C expression or the non-growth condition could affect the level of tolerance, high-density (∼1×10^9^ cells/ml) or low-density (∼1×10^6^ cells/ml) stationary phase *B. subtilis* cells were analyzed. An overnight culture of BG689 cells grown in MMS7 was washed with fresh, pre-warmed MMS7, and resuspended to ∼1×10^9^ cells/ml, and then exposed to 0.5% Xyl for 120 or 240 min to induce ζY83C toxin expression (Figure 2A and 2B). Expression of the ζY83C toxin under these growth conditions rendered a subpopulation of cells tolerant of ζY83C toxin action (2–6×10^−4^ survivals, Figure 2A and 2B), which is 5- to 10-times more tolerant of toxin action when compared to moderate-density exponentially growing cells (Figure 1A and 1B). The nature of ζ toxin tolerance in high-density stationary phase cells is unknown. Under this condition, the expression of host-encoded toxins (e.g., RtbD, RtbG, RtbI, RtbL, RtbN and/or SpoIISA) [40] and/or production of natural antimicrobials [41] could contribute to ζ toxin tolerance. Alternatively, in stationary phase low levels of translation could reduce the levels of ζY83C toxin. To test this hypothesis an overnight culture of BG689 cells was washed with fresh, pre-warmed MMS7, resuspended to ∼1×10^6^ cells/ml, and then exposed to 0.5% Xyl for 120 or 240 min to induce ζY83C toxin expression (Figure 2C and 2D). Expression of the toxin significantly reduced the tolerant fraction (6–8×10^−5^ survivals) to levels comparable to moderate-density exponentially growing BG689 cells (Figure 1A and 1B), suggesting that toxin expression was marginally affected if at all in stationary phase, and that the number of ζY83C toxin tolerant cells does not depend on the growth stage.

Transient addition of Amp, Cip or Tri to high-density slow- or non-growing cells increased the rate of tolerance 100- to 10^6^-fold after 120 or 240 min (Figure 2A and 2B) in comparison with exponentially growing moderate-density cells (Figure 1A and 1B). It could be hypothesized that high-density non-growing cells may have a heterogeneously increased frequency of pre-existing cells refractory to the antimicrobial treatment (*e.g.*, the antimicrobials have their targets inactive, as might be the case upon Amp addition). Alternatively, high-density non-growing cells may be “drug indifferent” because no correction by per-cell in the antimicrobial concentration was performed. To address whether this large increment in antimicrobial-tolerance observed with high-density non-growing cells is an effect of lower per-cell antimicrobial concentration, stationary phase cells were diluted into fresh, pre-warmed MMS7 medium to ∼1×10^6^ cells/ml. The subpopulation of low-density non-growing cells persistant of transient exposure to an antimicrobial (Figure 2C and 2D) was smaller than in high-density non-growing cultures (Figure 2A and 2B), and more similar to moderate-density exponentially growing cells (see Figure 1A and 1B). It is likely that the dramatic increase in tolerance (up to 1000-fold) to antimicrobials (except Ery) in high-density non-growing cells might not be attributable to the refractoriness of high-density non-growing cells to antimicrobials, and it might reflect a per-cell antimicrobial concentration. It is worth mentioning that MICs were estimated using low-density cells (1 to 3×10^6^ cells/ml for 16 h, at 37°C).

In stationary phase *E. coli* cells, HipA7 (over)expression exhibits an elevated survival rate (100- to 10,000-fold) upon Amp addition in non- or extremely slow-growing cells [3], [5], [6]. We addressed whether low-density growth-arrested cells expressing the ζ toxin became more tolerant of antimicrobials. Expression of the ζY83C toxin and addition of Amp, Ery, Cip or Tri significantly reduced the persistant fraction (1×10^−4^–7×10^−5^ survivals) of low-density stationary phase cells (Figure 2C and 2D) to levels comparable to moderate-density exponentially growing BG689 cells (Figure 1A and 1B). It is likely that first hypothesis (see above) might not apply upon ζY83C toxin expression, at least with the antimicrobials used, because in growth-arrested cells at the time of exposure to the antimicrobials the targets were not inactive.

### Antitoxin ε_2_ only reverses the effect of ζ in exponential or stationary phase cells exposed to antimicrobials

To rule out any specific contribution of the Y83C mutation in ζ toxin action, and to test whether the exit of dormancy, by expression of the ε_2_ antitoxin, contributes to antimicrobial sensitivity, the effect of induction of the long-living wt ζ toxin, at or near physiological concentrations (1,700 monomers/cell) (see Materials and methods), for 120 or 240 min, in exponentially moderate-density growing cells (∼5×10^7^ cells/ml) was analyzed. Here, wt ζ toxin expression was induced by addition of 1 mM IPTG, and ε_2_ antitoxin expression by 0.5% Xyl addition. The plasmid-borne ε gene (pCB799) confers resistance to Ery, thus the analysis of this antimicrobial was omitted [20].

Expression of ζ toxin for 120 or 240 min induced dormancy of the cell population, and a fraction was tolerant of ζ toxin action (∼4×10^−5^) (Figure 3A and 3B, “No AM”). Subsequent expression of the ε_2_ antitoxin, upon plating on LB agar containing 0.5% Xyl, led to partial exit of the dormant state, and CFUs increased ∼250-fold (Figure 3A and 3B, “No AM”). Alternatively the levels of ε_2_ antitoxin expression were not sufficient to overcome the effect of the wt ζ toxin. A full recovery was only observed in cells that were incubated with 0.5% Xyl for 120 min before plating in the presence of Xyl (see [20]). When the toxin tolerance of this strain was compared with the ζY83C strain, it was observed that the rate of survivals upon antimicrobial treatment was 2- to 6-fold lower in the former (Figure 3A) than in the latter strain (Figure 1A), suggesting that the leakage of the *P_hsp_* promoter or the longer half-life of the wt ζ toxin might contribute to reducing the survival fraction.

The transient addition of Amp, Cip or Tri decreased colony formation rendering 8×10^−4^ to 3×10^−7^ survivals after 120 min (Figure 3A). Similar rate of survivals were observed for cells only treated with the antimicrobial, and for cells treated with both antimicrobials and toxin and then induced ε_2_ antitoxin expression, suggesting that the ε_2_ antitoxin only reversed the toxin action. Expression of ζ toxin markedly decreased the antimicrobial tolerant fraction to the limit of detection (Figure 3A and 3B), suggesting that the exposure to both, an antimicrobial and ζ toxin, markedly increased the efficacy of the antimicrobials, leading to MDS.

To test whether expression of ε_2_ antitoxin also reversed the effect of ζ-mediated dormancy in high-density non-growing cells, early stationary phase BG1125 cells (∼1×10^9^ cells/ml) were analyzed. Expression of ζ toxin induced dormancy of the cell population, but a fraction of high-density cells was tolerant (∼2×10^−5^) to ζ toxin action (Figure 3C and 3D), which is a value similar to the one obtained in exponential growth. Expression of ζ toxin and treatment with Cip (Figure 3C and 3D) and Tri (data not shown) significantly reduced CFU of BG1125 cells, leading to MDS. The expression of ε_2_ antitoxin reversed the effect of the ζ toxin (Figure 3C and 3D). It is likely that: i) ζ toxin induces a reversible dormant state rather than cell killing; ii) there are many distinct subpopulations of tolerant cells (Figure 3); and iii) toxin and antimicrobial tolerants are different subpopulations of cells, because expression of ε_2_ antitoxin reversed only the effect of the ζ toxin.

### The absence of RelA enhances tolerance to both ζY83C and an antimicrobial

In bacteria, one rapid and sophisticated response to nutrient limitation is the accumulation of (p)ppGpp, which induces a global response to environmental stress, and is the major determinant of growth rate control [24]. The cellular response to ζ toxin expression resembles the nutrient starvation response, since toxin expression inhibits DNA, RNA and phospholipid synthesis, decreases the ATP and GTP pools, and increases (p)ppGpp [20]. Hyper-tolerance to ζ toxin action was observed in *B. subtilis* Δ*relA* cells that lack the major (p)ppGpp synthase [20]. Similarly, vancomycin hyper-tolerance is observed in *Enterococcus faecalis* Δ*relA* cells [42]. Conversely, in *E. coli* cells high levels of *hipA*7 diminished the levels of persistence in Δ*relA* cells when compared to the wt context [21], [22], [43]. How can we rationalize this apparent contradiction? In Firmicutes the intracellular levels of (p)ppGpp are maintained by the bifunctional RelA, which both synthesizes and degrades (p)ppGpp in response to the cellular nutritional status, and by one or two secondary monofunctional synthases (SasA and SasB) (SI Annex S1 in file S1) [27], [28], [44]. The role of these monofunctional synthases is to fine-tune any downward levels of (p)ppGpp during homeostatic growth of wt cells (SI Annex S1 in file S1, Figure S1 in file S2) [20], [25], [27]–[29], [44], so that in the Δ*relA* context, there are “dysregulated or uncontrolled” low undetectable (p)ppGpp levels [27], [28] because there is a low continuous (p)ppGpp synthesis, by the contribution of the SasA and SasB synthases, that cannot be hydrolyzed in the absence of RelA (see SI Annex S1 in file S1).

To test whether these “uncontrolled” basal (p)ppGpp levels might contribute also to antimicrobial hyper-tolerance we analyzed the effect of different antimicrobials in the presence or absence of toxin expression in the Δ*relA* context (Figure 4). Exponentially growing ∼5×10^7^ Δ*relA* cells/ml were treated with Xyl or transiently exposed to different antimicrobials. As previously observed, the absence of RelA rendered exponentially growing cells ∼100-fold more tolerant of ζY83C toxin action (hyper-tolerance) (Figure 4A and S3A) when compared to *relA* ^+^ cells (Figure 1). After transient exposure to Amp-, Cip- or Tri-treatment for 120 or 240 min, the surviving fraction (∼2×10^−1^ to ∼4×10^−4^, Figure 4A and S3A) was markedly increased when compared to the survival rate observed in *relA* ^+^ cells (∼2×10^−2^ to ∼4×10^−7^) (Figure 1A). Expression of ζ toxin and treatment with the different antimicrobials reduced CFU to levels comparable to toxin alone (Figure 4A and Figure S3A in file S2).

When high-density non-growing Δ*relA* cells (∼1×10^9^ cells/ml) were transiently exposed to both ζY83C toxin and any of the antimicrobials, the rate of non-inheritable tolerance increased 1,000- to 5,000-fold (Figure S3B and S3C in file S2) when compared to high-density non-growing *relA* ^+^ cells (Figure 2A and 2B). These results suggest that the Δ*relA* mutation confers a MDT phenotype. In contrast, (p)ppGpp accumulation correlates with AM persistance in proteobacteria (SI Annex S1 in file S1), and overexpression of the HipA7 toxin facilitates the development of Amp persisters through the production of (p)ppGpp [21], [22]. It was reported that the stringent response in *P. aeruginosa* facilitates persister formation in stationary phase cells by controlling the levels of reactive oxygen species [31], [45], raising the hypothesis that persistence depends on factors that regulate the lethal effect of reactive oxygen species. Unlike in *P. aeruginosa* cells, reactive oxygen species do not contribute to ζ toxin tolerance (SI Annex S1 in file S1).

### General stress response does not contribute to antimicrobial tolerance

Toxin and antimicrobial hyper-tolerance, in the Δ*relA* context, can also be attributed to the absence of the general stress response, because the strain used is impaired in *sigB* expression, and lacks the active σ^B^ general stress response regulator (SI Annex S2 in file S1). To test this hypothesis, we constructed a new set of strains in a *sigB* ^+^ background (Table S1 in file S2). A similar outcome to toxin and antimicrobial tolerance was observed when the Δ*relA sigB^−^* and Δ*relA sigB* ^+^ strains were compared (see Figure 4A and 5A), suggesting that the general stress response does not seem to be involved in toxin and antimicrobial hyper-tolerance. It is likely that the third hypothesis (see above) might not apply upon ζY83C toxin expression, at least with the antimicrobials used, because in the absence of stringent response (Δ*relA sigB* ^+^) or general stress response (Δ*relA sigB^−^*) hyper-tolerant cells were observed.

### Artificial reduction of (p)ppGpp levels decreases antimicrobial hyper-tolerance

It has been observed that the poor growth phenotype of *relA* cells can be suppressed by further decreasing the (p)ppGpp levels by impairment of the synthase domain of the bifunctional synthase-hydrolase RelA, or by the deletion of the SasB and/or SasA synthases [27], [28]. We hypothesized that “dysregulated” basal levels of (p)ppGpp by its “uncontrolled” synthesis by the SasA and/or SasB synthases might contribute to toxin and antimicrobial hyper-tolerance (see Figure 4A). To test this hypothesis we have taken advantage of relacin [46]. Relacin is a novel ppGpp analogue that poisons the active center of the (p)ppGpp synthases *in vitro*, and reduces (p)ppGpp production in *B. subtilis* cells *in vivo* [46]. To evaluate whether the decrease of (p)ppGpp levels in the Δ*relA* context reduces the level of hyper-tolerance of toxin or antimicrobials, exponentially growing cells were pre-treated with a limited relacin concentration (1 mM) that shows no apparent effect on the proliferation of wt cells. When the cells reached ∼5×10^7^ cells/ml, 0.5% Xyl and/or Amp were added and the proportion of surviving cells after 120 min was analyzed (Figure 4B). When Δ*relA* cells were pre-treated with relacin the surviving fraction of toxin, Amp or both treated cultures significantly decreased to levels comparable to *relA* ^+^ cells (compare Figure 1A and 4B). It is likely that: i) transient addition of relacin is sufficient to overcome the hyper-tolerance phenotype observed in the Δ*relA* context; ii) relacin might interact with the active center of SasA and/or SasB synthases and poison (p)ppGpp production; and iii) an artificial decrease in basal (p)ppGpp levels is sufficient to overcome the hyper-tolerance phenotype observed in the Δ*relA* context.

### Reduction of basal (p)ppGpp levels contributes to reducing antimicrobial hyper-tolerance

To test the contribution of each synthase toward the dysregulated low undetectable (p)ppGpp levels responsible for toxin and antimicrobial hyper-tolerance, strains lacking one or more synthases in a *sigB* ^+^ background were constructed and analyzed. Moderate-density exponentially growing Δ*relA*, Δ*sasA*, Δ*sasB*, Δ*sasA* Δ*sasB*, Δ*relA* Δ*sasB*, Δ*relA* Δ*sasA* and Δ*relA* Δ*sasA* Δ*sasB* (∼5×10^7^ cells/ml) cells were transiently exposed to Xyl, Amp or both for 120 min and the rate of tolerance analyzed (Figure 5A, 5B and S4). Three different outcomes were observed. First, an increased phenotypic hyper-tolerance to the toxin or to the antimicrobial or to both was observed in the Δ*relA* context (Figure 5A). Second, in the absence of SasA or RelA and SasA an intermediate phenotype was observed (Figure S4 in file S2). Here, the cells were slightly more tolerant of the toxin or Amp than wt cells, but less tolerant that in the Δ*relA* context. Third, in the absence of RelA and SasB or RelA, SasA and SasB a significant decrease in toxin and Amp tolerance was observed (Figure 5A and 5B), confirming that the absence of RelA alone is not involved in toxin and antimicrobial hyper-tolerance. It is likely that (p)ppGpp synthesized by SasB, and in minor extent by SasA, contributes to hyper-tolerance in the *relA* context. Conversely, in Proteobacteria (*e.g.*, *E. coli* and *P. aeruginosa*) dysregulated high (p)ppGpp levels lead to hyper-tolerance [31], [47]–[49]. In *E. coli* cells, artificial overexpression of the *relA* gene or in the *spoT*1 context (with attenuated hydrolase activity), lead to high dysregulated (p)ppGpp levels and hyper-tolerance [48], [49].

### Dysfunction of GTP homeostasis contributes to eradicating antimicrobial tolerance

In the presence of low (p)ppGpp (Δ*relA* Δ*sasB*) or in its absence, ([p]ppGpp^0^, Δ*relA* Δ*sasA* Δ*sasB*), GTP levels increase (Figure S1 in file S2) [28], [44]. Since the intracellular GTP pool is markedly increased in the Δ*relA* Δ*sasB* or Δ*relA* Δ*sasA* Δ*sasB* context, even in the presence of ζY83C toxin expression (Figure S1 in file S2, our unpublished results), we hypothesized that it is the dysregulation of the GTP pool that could lead to the observed lethality upon exposure to the toxin and Amp (see SI Annex S1 in file S1). To test this hypothesis (see [44]), we artificially lowered the GTP pool by addition of decoyinine, which is a GMP synthetase inhibitor [44], [50]. Exponentially growing Δ*sasB* Δ*relA* cells were pre-treated with decoyinine (100 µg/ml), and when the cells reached moderate-density, ∼5×10^7^ cells/ml, toxin expression was induced and/or Amp was added, and survival was analyzed 120 min later (Figure 5B).

The artificial reduction of the GTP (or GDP) levels by the transient addition of decoyinine increased the rate of survival of Δ*sasB* Δ*sasA* Δ*relA* ([p]ppGpp^0^) and Δ*sasB* Δ*relA* (low [p]ppGpp) (Figure 5B, data not shown). However, when *relA* ^+^ cells were exposed to the transient addition of decoyinine, no significant difference with the untreated control was observed [20]. It is likely that the fourth hypothesis (see above) might apply upon ζY83C toxin expression, because cells have evolved mechanisms to survive changing environments.

### Conclusions

We report here that in the presence of antimicrobials with different modes of action, ζ toxin expression, independently of the growth phase and the growth rate, alters the physiological mechanisms used by the cells to evade antimicrobial lethality and potentiates cell killing in wt context. Subsequent expression of the ε_2_ antitoxin specifically reverses ζ-induced dormancy, but not the persistence of the different antimicrobials, suggesting the presence of different subpopulations of tolerant cells. The use of wt ζ-GFP or inactive ζK46A-GFP fused variants (see [19]), in a follow up study could shed more light on the presence of these proposed subpopulations.

Different antimicrobials trigger specific responses that might have adaptive values [51], and antimicrobial tolerance is due to many different mechanisms [52]. The molecular mechanism of tolerance might differ between bacteria of the γ-Proteobacteria class and those of the Firmicutes phylum due to differences in their cellular metabolism. In the former bacterial class, the monofunctional RelA synthase is required for persistence, (p)ppGpp functions as a signal that determines whether single cells differentiate into a persistent state, and the persistence level increases in the presence of high uncontrolled (p)ppGpp levels [31], [47]–[49]. In bacteria of the Firmicutes phylum, inactivation of the bifunctional RelA synthase, and the presence of low uncontrolled (p)ppGpp levels leads to hyper-tolerance of toxin and/or of antimicrobials (Figure 4 and 5). It is likely that (p)ppGpp homeostasis contributes to persistence, but *E. coli* and *B. subtilis* cells use (p)ppGpp in different ways to survive starvation and the mode of action of these secondary messengers is significantly different between *E. coli* [48], [49] and *B. subtilis* (this work) [20]. In *B. subtilis*, (p)ppGpp directly regulates GTP homeostasis and GTP levels are critical for fitness (Fig. 5) [44]. The interplay between ζ-induced dormancy and the regulation of the (p)ppGpp and GTP levels can provide a rational to understand the molecular mechanisms of antimicrobial tolerance in Firmicutes. The active response to nutrient limitation, or increased (p)ppGpp and decreased GTP levels by ζ toxin expression, does not increase accumulation of tolerant cells [20]. Toxin-induced and host-controlled subtle changes in the threshold levels of (p)ppGpp and GTP, lead to three different outcomes: hyper-tolerance in Δ*relA* cells, normal tolerance in the wt and Δ*sasA* Δ*sasB* backgrounds, and increased cell death in the Δ*relA* Δ*sasB* or Δ*relA* Δ*sasA* Δ*sasB* context upon toxin expression or antimicrobial addition (SI Annex S1 in file S1, Figure S1 in file S2).

Is the active response to starvation different in the different bacterial genera? In *E. coli* (the best-characterized representative from the γ-Proteobacteria class) a mutant equivalent to *B. subtilis* Δ*relA*, which should be defective in the bifunctional synthase-hydrolase (*E. coli* SpoT) and proficient in the monofunctional synthase (*E. coli* RelA), was not viable [53]. In contrast, deletion of *spoT* did not cause a lethal phenotype in *P. aeruginosa* (another species of the γ-Proteobacteria class). Here, “dysregulation” of the (p)ppGpp levels, by lack of the hydrolase activity, renders high (p)ppGpp levels and cells hyper-tolerant of Cip [47]. Similarly, high unregulated (p)ppGpp levels, by artificial overexpression of the RelA gene or in the *spoT*1 context (attenuated hydrolase activity), lead to hyper-tolerance in *E. coli* cells [48]. Conversely, in *B. subtilis* Δ*relA* cells hyper-tolerance to ζ toxin and to different antimicrobials is observed in the presence of undetectable “dysregulated” (p)ppGpp levels. Similarly, vancomycin hyper-tolerance is enhanced *in Enterococcus faecalis* Δ*relA* cells (SI Annex S1 in file S1) [42]. This hyper-tolerant phenotype can be partially reversed by inactivation of either the SasA or the SasB synthase (Figure 5 and Figure S4) or artificial reduction of the (p)ppGpp pool by the presence of limiting relacin concentrations (Figure 4B). This finding, which underscores an all-or-nothing phenomenon, was ascribed to an imbalance of the (p)ppGpp pools.

A manifest loss of viability by the combined action of an antimicrobial and the toxin was observed in the presence of low (p)ppGpp or (p)ppGpp^0^ with a concomitant increase in GTP levels (Figure 5 and Figure S1 in file S2). An artificial decrease in *de novo* GTP synthesis in the presence of decoyinine, however, significantly decreases the sensitivity of the cells to the action of the ζ toxin or an antimicrobial in the Δ*relA* Δ*sasB* context (Figure 5B). Similar results were observed in the (p)ppGpp^0^ background (data not shown). The mechanism underlying cell death by an elevated GTP level remains poorly understood (see [44]), but we show here that this contributes to death by different antimicrobials and ζ toxin. The rheostat control of (p)ppGpp and GTP warrants further investigation at the genetic and biochemical levels, because it is conceivable that by modulating (p)ppGpp levels, as a new therapeutic strategy, we might inadvertently increase the burden of nosocomial infections, as in the absence of RelA, before attaining cell killing as in the (p)ppGpp^0^ context.

## Supporting Information

File S1 **Annex S1.** The alarmone (p)ppGpp mediates stress responses and controls toxin and antimicrobial tolerance in Firmicutes. **Annex S2** General stress response is not involved in toxin and antimicrobial tolerance.
(DOCX)

File S2 **Figure S1.** Schematic diagrams showing the pathway for stringent response in different genetic backgrounds of *B. subtilis*. The stringent control is induced in response to a number of stresses, and then the alarmone synthases synthesize (p)ppGpp by phosphorylation of GTP (GDP) in the presence of ATP. In response to stress by amino acid starvation (e.g., by addition of arginine hydroxamate) the levels of (p)ppGpp transiently increase >100-fold and the GTP pool decreases 50- to 60 fold in the wt or *sasA sasB* context [20], [25], [26]–[28]. Accumulation of (p)ppGpp produces transient and reversible inhibition of GTPases (e.g. Obg), affects nucleotide and lipid metabolism, *etc.*, and causes a halt in cell proliferation, by inhibiting DNA replication (DnaG), and normal tolerance to antimicrobial and to the ζ toxin. Cells exit the growth arrest upon reallocation of resources. In the *relA* context, the uncontrolled undetectable levels of (p)ppGpp lead to poor stress survival, but to antimicrobial and ζ toxin hyper-tolerance (see Figure 4). Toxin expression and Amp addition decrease the survival rate of Δ*sasA* Δ*sasB* Δ*relA* cells and to a minor extent of *sasB* Δ*relA* cells, and this effect is partially overcome when GTP synthesis is inhibited by decoyinine addition, suggesting that low GTP levels are necessary for tolerance. ^a^ND, not detected, assigned an arbitrary value of <1 in the wt unstressed context (10–20 µM), and increased to 1–3 mM after 10 min exposure to arginine hydroxamate [25], [26], [27], [28], [44]. ^b^The GTP levels are given relative to the values in the wt strain under unstressed conditions (∼5 mM), which are denoted by an arbitrary value of 100, and decreased to 80–100 µM after 10 min exposure to arginine hydroxamate [28], [44], [54]. ^c^In the absence of RelA, the addition of branched chain amino acids was required for cell growth. Figure S2. Efficacy of ampicillin and ciprofloxacin during exponential growth. BG689 cells were grown in MMS7 at 37°C up to ∼5×10^7^ cells/ml, then Amp, Cip or both antimicrobial were added. The cultures were incubated for 120 min and then plated onto LB agar plates. The symbols, the plating conditions, and the antimicrobial concentrations were those indicated in Figure 1. The number of CFUs relative to the non-induced/non-AM treated control is shown. Error bars show 95% confidence intervals of more than three independent experiments. (TIF). Figure S3 RelA is required for ζY83C toxin enhanced efficacy to different antimicrobials. BG1145 (Δ*relA*)-borne ζY83C gene was induced by the addition of 0.5% Xyl. BG1145 cells were grown to ∼5×10^7^ cells/ml in MMS7. Then 0.5% Xyl and/or an antimicrobial were added, and the cultures were incubated for 240 min with agitation at 37°C (A). BG1145 cells were grown to early stationary phase and diluted into fresh pre-warmed MMS7 to ∼1×10^9^ cells/ml. Then 0.5% Xyl and/or an antimicrobial were added to these high-density non-growing cells, and the cultures were incubated for 120 min (B) or 240 min (C) with agitation at 37°C. Appropriated dilutions were then plated on LB agar, and incubated for 36 h at 37°C. The symbols, the plating conditions, and the antimicrobial concentrations were those indicated in Figure 1. Error bars show 95% confidence intervals of more than three independent experiments. Figure S4 Variations in the levels of (p)ppGpp alter the outcome of ζY83C- tolerance and Amp persistence. BG1205 (Δ*sasA*), BG1207 (Δ*sasB*), BG1211 (Δ*sasA* Δ*sasB*) or BG1301 (Δ*relA* Δ*sasA*) cells were grown in MMS7 to ∼5×10^7^ cells/ml, then 0.5% Xyl and/or Amp was added and the cultures were incubated for 120 min. Appropriate dilutions were then plated on LB agar and incubated for 36 h at 37°C. The symbols, the plating conditions, and the antimicrobial concentrations were those indicated in Figure 1. Error bars show 95% confidence intervals of more than three independent experiments. Table S1. Bacterial strains used.
(PDF)
