# Supplementary material for: Role of Toxin ζ and Starvation Responses in the Sensitivity to Antimicrobials
Source: PLoS One. 2014 Jan 29;9(1):e86615. doi: 10.1371/journal.pone.0086615 (PMC3906061; doi:10.1371/journal.pone.0086615)
Supplement: File S1 — Annex S1. The alarmone (p)ppGpp mediates stress responses and controls toxin and antimicrobial tolerance in Firmicutes. Annex S2 General stress response is not involved in toxin and antimicrobial tolerance. (DOCX) [file pone.0086615.s001.docx]

**Role of Toxin ζ and Starvation Responses in the Sensitivity to Antimicrobials**

**Mariangela Tabone, Virginia S. Lioy, Silvia Ayora, Cristina Machón, and Juan C. Alonso**

*Departamento de Biotecnología Microbiana, Centro Nacional de Biotecnología (CNB-CSIC), Madrid, Spain*

Supporting Information

**Annex S1.** *The alarmone (p)ppGpp mediates stress responses and controls toxin and antimicrobial tolerance in Firmicutes*

(p)ppGpp is produced from GTP/GDP and ATP, and its production during amino acid starvation is accompanied by a decrease in the GTP pool [[1](#_ENREF_1),[2](#_ENREF_2)]. Bacteria of the β- and γ-Proteobacteria classes possess a bifunctional SpoT enzyme that has both synthase and hydrolase activity, and a monofunctional synthase, RelA, that is the major contributor to the (p)ppGpp pool [[3](#_ENREF_3),[4](#_ENREF_4)]. Other bacteria have either a single bifunctional RelA-SpoT enzyme, named RelA, or a bifunctional RelA and one or two monofunctional synthases, with RelA being the major contributor to the (p)ppGpp pool [[3](#_ENREF_3),[4](#_ENREF_4)].

In *E. coli*, (p)ppGpp enables resistance to starvation by binding to RNA polymerase, in concert with DksA, and directly altering transcription of many genes, including rRNA genes [[3](#_ENREF_3),[4](#_ENREF_4)]. However, this mechanism does not apply in *B. subtilis*, in which (p)ppGpp does not affect RNA polymerase and DksA is not present [[5](#_ENREF_5)]. Here, (p)ppGpp regulates transcription indirectly via GTP [[2](#_ENREF_2)]. *In B. subtilis* wt cells, the (p)ppGpp level is inversely proportional to the growth mode, *i.e*. slow growth or survival mode leads to maximal levels, suboptimal growth leads to an elevated level, and normal growth leads to low (p)ppGpp levels (Figure S1) [[3](#_ENREF_3),[4](#_ENREF_4)]. The rapid accumulation of (p)ppGpp during stress conditions is dependent on the RelA enzyme [[6](#_ENREF_6),[7](#_ENREF_7)]. In exponentially growing, unstressed, wt *B. subtilis* cells, the levels of (p)ppGpp are at the limit of detection (10 – 20 μM) [[2](#_ENREF_2)]. They are below the level of detection in the Δ*relA* strain, and even lower in the Δ*relA* Δ*sasA* or Δ*relA* Δ*sasB* mutant strains (Figure S1) (our unpublished results) [[6](#_ENREF_6),[7](#_ENREF_7),[8](#_ENREF_8)].

Active starvation responses in *E. coli* cells switch the cell metabolism from the growth mode to the survival mode, resulting in slow growth, high (p)ppGpp levels, inhibition of peptidoglycan metabolism, down-regulation of transcriptional levels, and antimicrobial tolerance [[9-17](#_ENREF_9)]. Furthermore, high unregulated (p)ppGpp levels contribute to antimicrobial persitence [[18-20](#_ENREF_18)]. Unlike *E. coli* cells where deficiency in (p)ppGpp synthesis produces a reduced level of persisters [[14](#_ENREF_14)], disruption of RelA is pleotropic in *B. subtilis*, leading to basal “uncontrolled” [p]ppGpp levels, a ~3-fold reduction in the GTP pool, and poor growth but toxin and antimicrobial hyper-tolerance (Figure S1) [[21](#_ENREF_21)]. Both monofunctional synthases, SasA and SasB, should contribute to the accumulation of unknown (p)ppGpp levels in the Δ*relA* context (Figure S1) [[6](#_ENREF_6),[7](#_ENREF_7)]. However, the growth defect of Δ*relA* cells is restored and the hyper-tolerance reduced by inactivation of either the SasA or the SasB synthase (Figures 5 and S4) [[6](#_ENREF_6),[7](#_ENREF_7)]. It is likely, therefore, that in the absence of RelA, the SasA and/or SasB synthases constitutively synthesize (p)ppGpp leading to “uncontrolled or dysregulated” basal (p)ppGpp levels, reduced growth rate and toxin and antimicrobial hyper-tolerance (Figure S1) [[6-8](#_ENREF_6)].

In unstressed cells, the intracellular GTP levels are comparable in wt, Δ*sasA,* Δ*sasB* and Δ*sasA* Δ*sasB* cells, but decreased 2- to 3-fold in Δ*relA,* Δ*relA* Δ*sasB* and Δ*relA* Δ*sasA* cells (Figure S1) [[6](#_ENREF_6)]. Upon aminoacid starvation by addition of serine or arginine hydroxamate, the (p)ppGpp pool increases in the wt, Δ*sasA,* Δ*sasB* and Δ*sasA* Δ*sasB* strains, and under this condition the GTP pool decreases 50- to 60-fold (Figure S1) [[6](#_ENREF_6)]. Conversely, in Δ*relA* Δ*sasA*, Δ*relA* Δ*sasB* or Δ*relA* Δ*sasA* Δ*sasB* cells, (p)ppGpp accumulation was not detectable, and the GTP pool did not significantly increase in Δ*relA* Δ*sasA* cells, but it increased 2- to 3-fold in Δ*relA* Δ*sasB* and Δ*relA* Δ*sasA* Δ*sasB* cells (Figure S1) (our unpublished results) [[6](#_ENREF_6)]. These results suggest that one of the key roles of RelA is to regulate the amount of (p)ppGpp, which negatively controls the GTP levels [[2](#_ENREF_2)], and that the contribution of SasA and SasB is to fine-tune the intracellular pools of (p)ppGpp, and decrease the reserves of GTP [[6](#_ENREF_6),[7](#_ENREF_7)].

Previously it has been reported that the stringent response of *P. aeruginosa* facilitates persister formation in stationary phase by controlling reactive oxygen species (ROS) metabolism [[16](#_ENREF_16)]. However, It was shown that *rel*^+^ *B. subtilis* cells grown in the presence of the iron chelator 2,2’-dipyridyl, at concentrations that block the Fenton reaction *in vivo* without affecting the oxygen concentration [[22](#_ENREF_22)], did not show significant difference in plating efficiency after toxin and antimicrobial treatment when compared to the untreated control (our unpublished results) [[21](#_ENREF_21)]. It is likely that a decrease of endogenous oxidant production does not increase ζ toxin tolerance. This is consistent with the observation that the stringent response and the oxidative stress response show no obvious link at least in *B. subtilis* cells [[23](#_ENREF_23)]. Indeed, expression of the ζ toxin neither correlates with the accumulation of reactive oxygen species, with enhanced expression of stress envelop sigma factors (σ^M^, σ^X^, σ^W^) that alter the (p)ppGpp levels, with induction of the SOS response nor with increased expression of other chromosomal-encoded TA loci (see [[21](#_ENREF_21)]). However, we cannot rule out its influence because many AMs directly (as Cip addition) or indirectly (as Amp) increase ROS and (p)ppGpp levels, and induce the SOS response [[24-26](#_ENREF_24)].

Induction of the stringent response (by amino acid starvation) triggers a decrease in the GTP pool, which is involved in the repression of CodY [[27](#_ENREF_27)]. CodY is a branched chain amino acids- and GTP-sensing protein that functions as a global transcriptional regulator under high-GTP levels conditions [[27](#_ENREF_27)]. CodY triggers adaptation to starvation by secretion of proteases, the expression of amino acid transporters and the modulation of catabolic pathways [[28](#_ENREF_28)]. Although CodY is not directly involved in GTP biosynthesis, loss-of-function mutations in *codY* decrease GTP levels [[29](#_ENREF_29)].

**Annex S2.** *General stress response is not involved in toxin and antimicrobial tolerance*

In *E. coli* cells (p)ppGpp is required to induce the production of σ^S^ (ortholog of *B. subtilis* σ^B^) that in turn directs gene expression of persistence gene products [[12](#_ENREF_12)]. *B. subtilis* GTP-binding Obg protein, which belongs to the conserved small GTPase protein family, co-crystallizes with ppGpp (see [[4](#_ENREF_4)]). The mechanism by which Obg promotes growth is not clear. Obg interacts with several regulators necessary for the activation of σ^B^ (encoded by *sigB* gene), the global controller of the general stress regulon. The isogenic strains used in Figures 1 to 4 carried a mutation (termed *rsbV*37) that disables the general stress regulon under the control of the σ^B^ factor (see Table S1). To test the contribution of the general stress regulon to toxin or antimicrobial tolerance, the ζY83C toxin expression cassette (*P_xylA_*Y83C) was introduced as a single copy in the *amy* locus of *sigB*^+^ (BG1202) and *sigB*^+^ Δ*relA* (BG1203) strains (Table S1). Expression of ζY83C toxin or Amp addition led to a high rate of phenotypic tolerance/ persistence independent of the general stress regulon in the absence of RelA when compared to *relA*^+^ cells (Figure 5A). Similar results were observed in the *rsbV*37 background (Figure 4A), suggesting that the σ^B^ regulon did not affect toxin or antimicrobial tolerance (*sigB*^+^ versus *sigB*^-^). Unlike *B. subtilis* cells (Figure 5A), the intrinsic resistance of *E. coli* to various antimicrobial agents requires (p)ppGpp, the general stress regulon σ^S^ and DksA [[12](#_ENREF_12)]. In *B. subtilis* cells the presence of a gene function equivalent to *dksA* is not obvious, and the general stress regulon σ^B^ does not significantly contribute to tolerance.

**References**

1. Lopez JM, Dromerick A, Freese E (1981) Response of guanosine 5'-triphosphate concentration to nutritional changes and its significance for *Bacillus subtilis* sporulation. J Bacteriol 146: 605-613.

2. Kriel A, Bittner AN, Kim SH, Liu K, Tehranchi AK, et al. (2012) Direct regulation of GTP homeostasis by (p)ppGpp: a critical component of viability and stress resistance. Mol Cell 48: 231-241.

3. Cashel M, Gentry DR, Hernandez VJ, Vinella D (1996) The stringent response. In: Neidhardt FC, Curtiss III R, Ingraham JL, Lin ECC, Low KB et al., editors. *Escherichia coli* and *Salmonella typhimurium*: cellular and molecular biology, 2nd ed. Washington, DC: American Society for Microbiology. pp. 1458–1496.

4. Potrykus K, Cashel M (2008) (p)ppGpp: still magical? Annu Rev Microbiol 62: 35-51.

5. Krasny L, Gourse RL (2004) An alternative strategy for bacterial ribosome synthesis: *Bacillus subtilis* rRNA transcription regulation. EMBO J 23: 4473-4483.

6. Nanamiya H, Kasai K, Nozawa A, Yun CS, Narisawa T, et al. (2008) Identification and functional analysis of novel (p)ppGpp synthetase genes in *Bacillus subtilis*. Mol Microbiol 67: 291-304.

7. Srivatsan A, Wang JD (2008) Control of bacterial transcription, translation and replication by (p)ppGpp. Curr Opin Microbiol 11: 100-105.

8. Wendrich TM, Marahiel MA (1997) Cloning and characterization of a relA/spoT homologue from *Bacillus subtilis*. Mol Microbiol 26: 65-79.

9. Davis BD (1948) Isolation of biochemically deficient mutants of bacteria by penicillin. J Am Chem Soc 70: 4267.

10. Lederberg J, Zinder N (1948) Concentration of biochemical mutants of bacteria with penicillin. J Am Chem Soc 70: 4267.

11. Rodionov DG, Ishiguro EE (1995) Direct correlation between overproduction of guanosine 3',5'-bispyrophosphate (ppGpp) and penicillin tolerance in *Escherichia coli*. J Bacteriol 177: 4224-4229.

12. Greenway DL, England RR (1999) The intrinsic resistance of *Escherichia coli* to various antimicrobial agents requires ppGpp and sigma s. Lett Appl Microbiol 29: 323-326.

13. Pisabarro AG, De Pedro MA, Ishiguro EE (1990) Dissociation of the ampicillin-induced lysis of amino acid-deprived *Escherichia coli* into two stages. J Bacteriol 172: 2187-2190.

14. Korch SB, Henderson TA, Hill TM (2003) Characterization of the hipA7 allele of *Escherichia coli* and evidence that high persistence is governed by (p)ppGpp synthesis. Mol Microbiol 50: 1199-1213.

15. Pomares MF, Vincent PA, Farias RN, Salomon RA (2008) Protective action of ppGpp in microcin J25-sensitive strains. J Bacteriol 190: 4328-4334.

16. Nguyen D, Joshi-Datar A, Lepine F, Bauerle E, Olakanmi O, et al. (2011) Active starvation responses mediate antibiotic tolerance in biofilms and nutrient-limited bacteria. Science 334: 982-986.

17. Gerdes K, Maisonneuve E (2012) Bacterial persistence and toxin-antitoxin loci. Annu Rev Microbiol 66: 103-123.

18. Amato SM, Orman MA, Brynildsen MP (2013) Metabolic control of persister formation in Escherichia coli. Mol Cell 50: 475-487.

19. Maisonneuve E, Castro-Camargo M, Gerdes K (2013) (p)ppGpp Controls Bacterial Persistence by Stochastic Induction of Toxin-Antitoxin Activity. Cell 154: 1140-1150.

20. Viducic D, Ono T, Murakami K, Susilowati H, Kayama S, et al. (2006) Functional analysis of spoT, relA and dksA genes on quinolone tolerance in Pseudomonas aeruginosa under nongrowing condition. Microbiol Immunol 50: 349-357.

21. Lioy VS, Machon C, Tabone M, Gonzalez-Pastor JE, Daugelavicius R, et al. (2012) The zeta toxin induces a set of protective responses and dormancy. PloS ONE 7: e30282.

22. Imlay JA, Chin SM, Linn S (1988) Toxic DNA damage by hydrogen peroxide through the Fenton reaction *in vivo* and *in vitro*. Science 240: 640-642.

23. Eymann C, Homuth G, Scharf C, Hecker M (2002) *Bacillus subtilis* functional genomics: global characterization of the stringent response by proteome and transcriptome analysis. J Bacteriol 184: 2500-2520.

24. Kohanski MA, Dwyer DJ, Hayete B, Lawrence CA, Collins JJ (2007) A common mechanism of cellular death induced by bactericidal antibiotics. Cell 130: 797-810.

25. Cirz RT, Jones MB, Gingles NA, Minogue TD, Jarrahi B, et al. (2007) Complete and SOS-mediated response of *Staphylococcus aureus* to the antibiotic ciprofloxacin. J Bacteriol 189: 531-539.

26. Miller C, Thomsen LE, Gaggero C, Mosseri R, Ingmer H, et al. (2004) SOS response induction by beta-lactams and bacterial defense against antibiotic lethality. Science 305: 1629-1631.

27. Ratnayake-Lecamwasam M, Serror P, Wong KW, Sonenshein AL (2001) *Bacillus subtilis* CodY represses early-stationary-phase genes by sensing GTP levels. Genes & Dev 15: 1093-1103.

28. Sonenshein AL (2007) Control of key metabolic intersections in *Bacillus subtilis*. Nature Rev Microbiol 5: 917-927.

29. Molle V, Nakaura Y, Shivers RP, Yamaguchi H, Losick R, et al. (2003) Additional targets of the *Bacillus subtilis* global regulator CodY identified by chromatin immunoprecipitation and genome-wide transcript analysis. J Bacteriol 185: 1911-1922.

30. Bennett BD, Kimball EH, Gao M, Osterhout R, Van Dien SJ, et al. (2009) Absolute metabolite concentrations and implied enzyme active site occupancy in *Escherichia coli*. Nature Chem Biol 5: 593-599.
