# Supplementary material for: Role of Toxin ζ and Starvation Responses in the Sensitivity to Antimicrobials
Source: PLoS One. 2014 Jan 29;9(1):e86615. doi: 10.1371/journal.pone.0086615 (PMC3906061; doi:10.1371/journal.pone.0086615)
Supplement: File S2 — Figure S1. Schematic diagrams showing the pathway for stringent response in different genetic backgrounds of B. subtilis. The stringent control is induced in response to a number of stresses, and then the alarmone synthases synthesize (p)ppGpp by phosphorylation of GTP (GDP) in the presence of ATP. In response to stress by amino acid starvation (e.g., by addition of arginine hydroxamate) the levels of (p)ppGpp transiently increase >100-fold and the GTP pool decreases 50- to 60 fold in the wt or sasA sasB context [20], [25], [26]–[28]. Accumulation of (p)ppGpp produces transient and reversible inhibition of GTPases (e.g. Obg), affects nucleotide and lipid metabolism, etc., and causes a halt in cell proliferation, by inhibiting DNA replication (DnaG), and normal tolerance to antimicrobial and to the ζ toxin. Cells exit the growth arrest upon reallocation of resources. In the relA context, the uncontrolled undetectable levels of (p)ppGpp lead to poor stress survival, but to antimicrobial and ζ toxin hyper-tolerance (see Figure 4). Toxin expression and Amp addition decrease the survival rate of ΔsasA ΔsasB ΔrelA cells and to a minor extent of sasB ΔrelA cells, and this effect is partially overcome when GTP synthesis is inhibited by decoyinine addition, suggesting that low GTP levels are necessary for tolerance. aND, not detected, assigned an arbitrary value of <1 in the wt unstressed context (10–20 µM), and increased to 1–3 mM after 10 min exposure to arginine hydroxamate [25], [26], [27], [28], [44]. bThe GTP levels are given relative to the values in the wt strain under unstressed conditions (∼5 mM), which are denoted by an arbitrary value of 100, and decreased to 80–100 µM after 10 min exposure to arginine hydroxamate [28], [44], [54]. cIn the absence of RelA, the addition of branched chain amino acids was required for cell growth. Figure S2. Efficacy of ampicillin and ciprofloxacin during exponential growth. BG689 cells were grown in MMS7 at 37°C up to ∼5×10^7^ cells/ [file pone.0086615.s002.pdf]

Role of Toxin ζ and Starvation Responses in the Sensitivity to Antimicrobials

Mariangela Tabone, Virginia S. Lioy, Silvia Ayora, Cristina Machón, and Juan C. Alonso

Departamento de Biotecnología Microbiana, Centro Nacional de Biotecnología (CNB-CSIC), Madrid, Spain

Supporting Information

Table S1. Bacterial strains used

Strain	Relevant mutant genotype	Reference
BG687[a]	+ *xylR*, P_{xylA}, *cat*	[21]
BG689[a]	+ *xylR*, P_{xylA}ζY83C *cat*	[21]
BG1145[a]	+ *xylR*, P_{xylA}ζY83C *cat*, Δ*relA:ery*	[21]
BG1125[a]	+ *lacI*, P_{hsp}ζ, *spc,* [pCB799, *xylR*, P_{xylA}ε, *cat*]	[21]
BG1127[a]	+ *lacI*, P_{hsp}, *spc,* [pCB799, *xylR*, P_{xylA}ε, *cat*]	[21]
BG1241[a]	+ *xylR*, P_{xylA} *cat*, Δ*mazF*	This work
BG1243[a]	+ *xylR*, P_{xylA}ζY83C *cat*, Δ*mazF*	This work
BG1202[b]	+ *xylR*, P_{xylA}ζY83C *cat*	This work
BG1203[b]	+ *xylR*, P_{xylA}ζY83C *cat*, Δ*relA:ery*	This work
BG1205[b]	+ *xylR*, P_{xylA}ζY83C *cat*, Δ*sasA:spc*	This work
BG1207[b]	+ *xylR*, P_{xylA}ζY83C *cat*, Δ*sasB*, *trpC2*	This work
BG1211[b]	+ *xylR*, P_{xylA}ζY83C *cat*, Δ*sasA:spc*, Δ*sasB*, *trpC2*	This work
BG1209[b]	+ *xylR*, P_{xylA}ζY83C *cat*, Δ*relA:ery*, Δ*sasB*, *trpC2*	This work
BG1301[b]	+ *xylR*, P_{xylA}ζY83C *cat*, Δ*relA:ery*, Δ*sasA:spc*	This work
BG1213[b]	+ *xylR*, P_{xylA}ζY83C *cat*, Δ*relA:ery*, Δ*sasA:spc*, Δ*sasB*, *trpC2*	This work

[a]The strains are isogenic with BG214 (*trpCE metA*5 *amyE1 ytsJ*1 *rsbV*37 *xre*1 *xkd*A1 att^{SPb} att^{ICEBs1}). [b]The strains are isogenic with PY79 (prototroph).

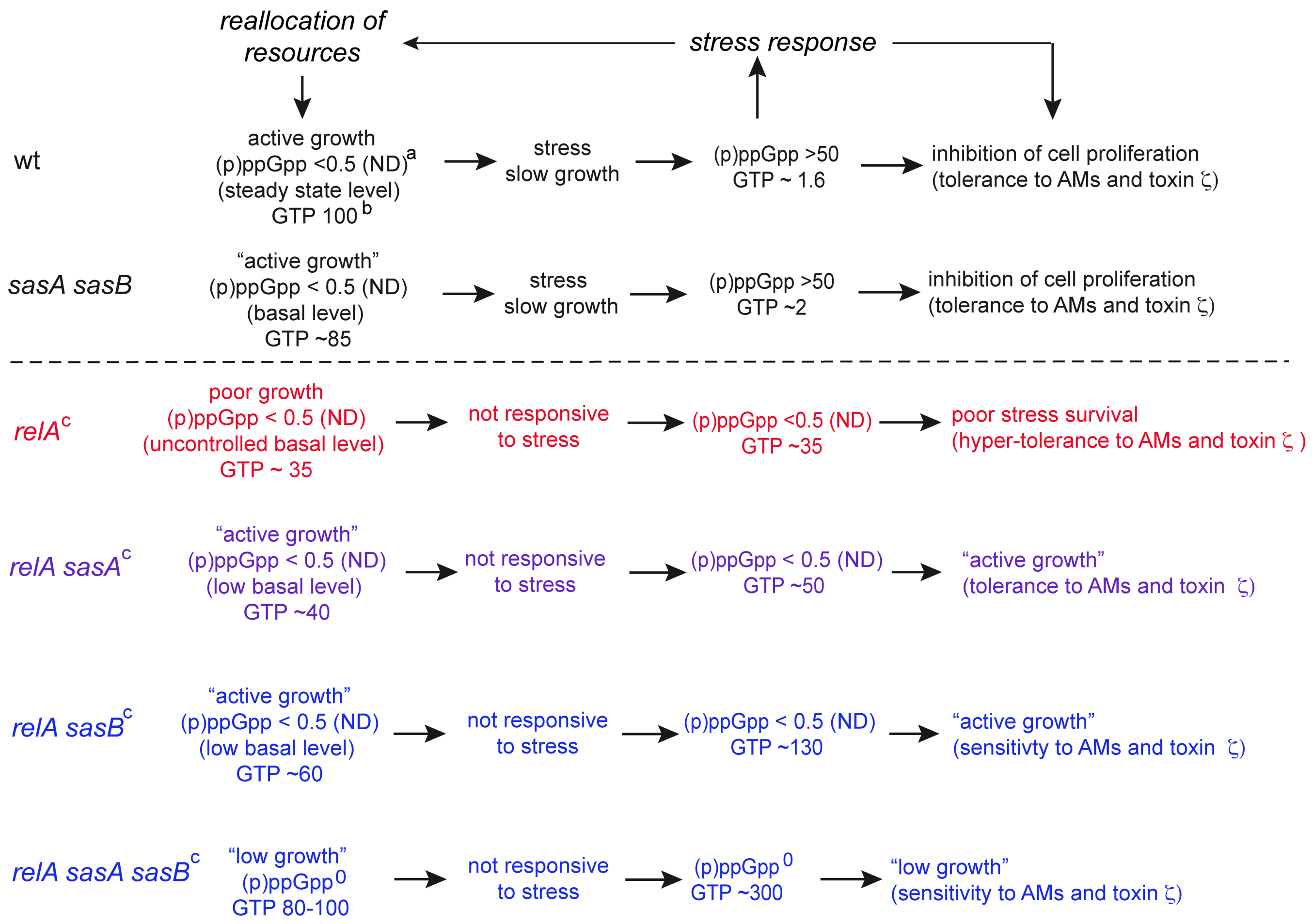

Figure S1. Schematic diagrams showing the pathway for stringent response in different genetic backgrounds of *B. subtilis*. The stringent control is induced in response to a number of stresses, and then the alarmone synthases synthesize (p)ppGpp by phosphorylation of GTP (GDP) in the presence of ATP. In response to stress by amino acid starvation (e.g., by addition of arginine hydroxamate) the levels of (p)ppGpp transiently increase >100-fold and the GTP pool decreases 50- to 60 fold in the wt or *sasA sasB* context [6-8,21,23]. Accumulation of (p)ppGpp produces transient and reversible inhibition of GTPases (e.g. Obg), affects nucleotide and lipid metabolism, *etc*., and causes a halt in cell proliferation, by inhibiting DNA replication (DnaG), and normal tolerance to antimicrobials and to the ζ toxin. Cells exit the growth arrest upon reallocation of resources. In the *relA* context, the uncontrolled undetectable levels of (p)ppGpp lead to poor stress survival, but to antimicrobial and ζ toxin hypertolerance (Figure 4). Toxin expression and Amp addition decrease the survival rate of Δ*sasA* Δ*sasB* Δ*relA* cells and to a minor extent of *sasB* Δ*relA* cells, and this effect is partially overcome when GTP synthesis is inhibited by decoyinine addition, suggesting that low GTP levels are necessary for tolerance. [a]ND, not detected, assigned an arbitrary value of <1 in the wt unstressed context (10 – 20 μM), and increased to 1-3 mM after 10 min exposure to arginine hydroxamate [2,6-8,23]. [b]The GTP levels are given relative to the values in the wt strain under unstressed conditions (~5 mM), which are denoted by an arbitrary value of 100, and decreased to 80 - 100 μM after 10 min exposure to arginine hydroxamate [2,6-8,30]. [c]In the absence of RelA, the addition of branched chain amino acids was required for cell growth.

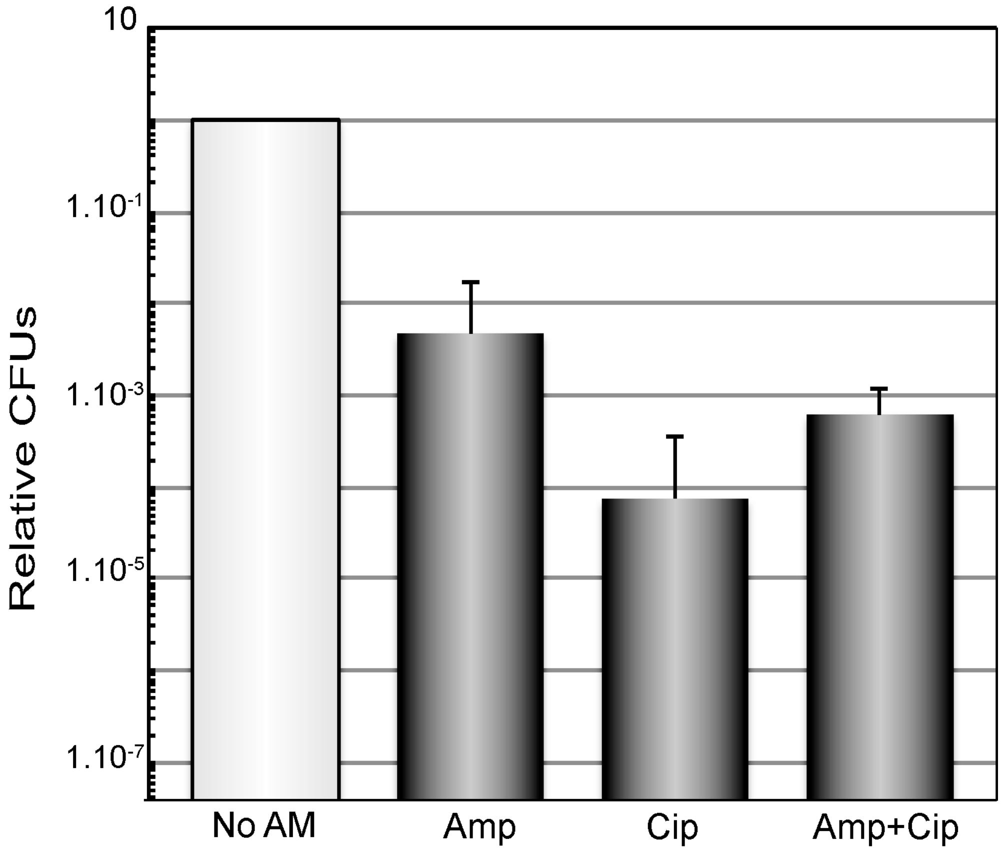

Figure S2. Efficacy of ampicillin and ciprofloxacin during exponential growth. BG689 cells were grown in MMS7 at 37ºC up to ~ 5 x 10^7 cells/ml, then Amp, Cip or both antimicrobial were added. The cultures were incubated for 120 min and then plated onto LB agar plates. The number of CFUs relative to the non-induced/non-AM treated control is shown. The symbols, the plating conditions, and the antimicrobial concentrations were those indicated in Figure 1. Error bars show 95% confidence intervals of more than three independent experiments.

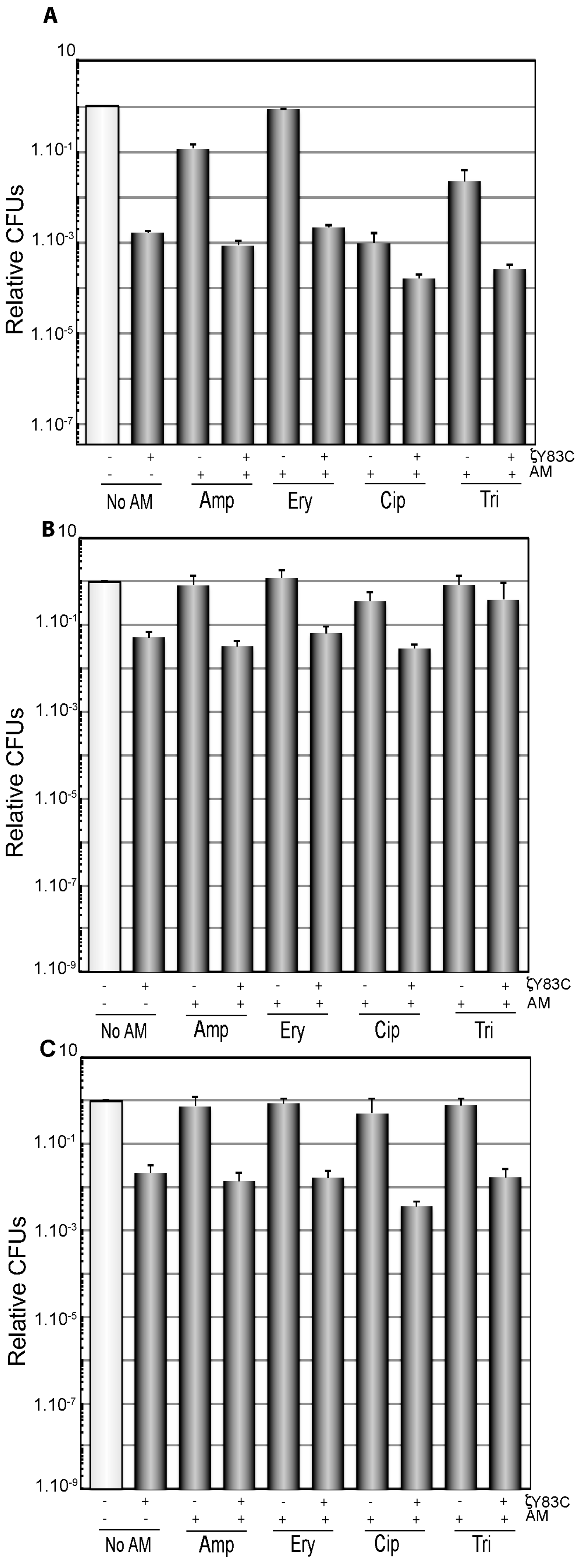

Figure S3. RelA is required for ζY83C toxin enhanced efficacy to different antimicrobials. BG1145 (Δ*relA*)-borne ζY83C gene was induced by the addition of 0.5% Xyl. BG1145 cells were grown to ~5 x 10^7 cells/ml in MMS7. Then 0.5% Xyl and/or an antimicrobial were added, and the cultures were incubated for 240 min with agitation at 37° C (A). BG1145 cells were grown to early stationary phase and diluted into fresh pre-warmed MMS7 to ~ 1x 10^9 cells/ml. Then 0.5% Xyl and/ or an antimicrobial were added to these high-density non-growing cells, and the cultures were incubated for 120 min (B) or 240 min (C) with agitation at 37° C. Appropriated dilutions were then plated on LB agar, and incubated for 36 h at 37° C. The symbols, the plating conditions, and the antimicrobial concentrations were those indicated in Figure 1. Error bars show 95% confidence intervals of more than three independent experiments.

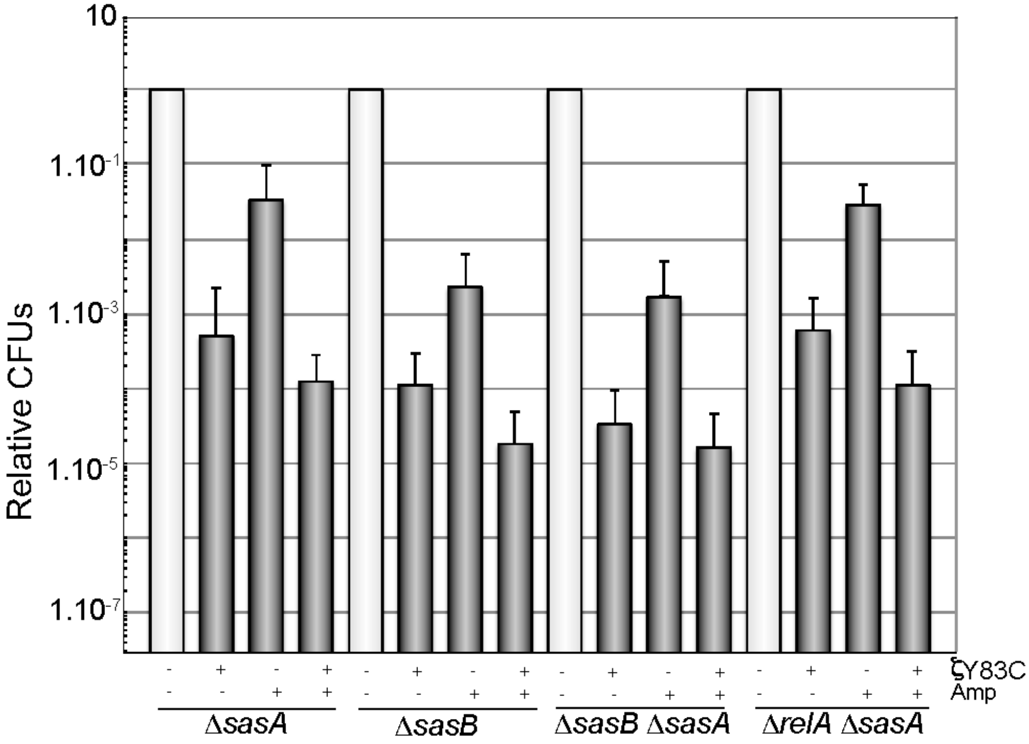

Figure S4. Variations in the levels of (p)ppGpp alter the outcome of ζY83C- tolerance and Amp persistence. BG1205 (Δ*sasA*), BG1207 (Δ*sasB*), BG1211 (Δ*sasA* Δ*sasB*) or BG1301 (Δ*relA* Δ*sasA*) cells were grown in MMS7 to ~5 x 10^7 cells/ml, then 0.5% Xyl and/or Amp was added and the cultures were incubated for 120 min. Appropriate dilutions were then plated on LB agar and incubated for 36 h at 37° C. The symbols, the plating conditions, and the antimicrobial concentrations were those indicated in Figure 1. Error bars show 95% confidence intervals of more than three independent experiments.
